# Relationship of vegetarianism with body weight loss and ASCVD

**DOI:** 10.3389/fnut.2024.1419743

**Published:** 2024-08-27

**Authors:** Yani Xu, Xuening Yang, Lina Yao, Yanping Liu, Panpan Hao

**Affiliations:** ^1^State Key Laboratory for Innovation and Transformation of Luobing Theory, The Key Laboratory of Cardiovascular Remodeling and Function Research, Chinese Ministry of Education, Chinese National Health Commission and Chinese Academy of Medical Sciences, Department of Cardiology, Qilu Hospital of Shandong University, Jinan, Shandong, China; ^2^School of Basic Medical Science, Cheeloo College of Medicine, Shandong University, Jinan, Shandong, China; ^3^Department of Radiology, Qilu Hospital of Shandong University, Jinan, Shandong, China

**Keywords:** vegan, vegetarianism, plant-based diet, obesity, ASCVD

## Abstract

**Introduction:**

The association between a plant-based diet and weight loss and atherosclerotic cardiovascular disease (ASCVD) has not been fully elucidated. We performed a pooled analysis and Mendelian randomization (MR) analysis to investigate this question.

**Methods:**

We searched for randomized controlled trials on the effects of a plant-based diet on weight loss compared with a non-plant-based diet. In addition, a two-sample MR study was conducted. IVs were obtained from the genome-wide association studies (GWAS) on the exposures, and we obtained summary statistics on the outcomes. The inverse-variance weighted (IVW) method was used as the main analysis and other MR methods were performed as supplementary analyses.

**Results:**

Individuals on the plant-based diet lost more weight than the non-plant-based diet group (WMD −0.96 kg; 95% CI: −1.32 to −0.60). Population conditions and energy restriction were identified as the study-level factors that influenced the pooling results in the subgroup analyses. Increased consumption of raw vegetables was significantly associated with lower BMI (IVW, β −0.35, 95% CI: −0.62 to −0.08, *p* = 0.012) and lower risk of obesity (IVW, OR 0.11, 95% CI: 0.01 to 0.99, *p* = 0.048), coronary heart disease (IVW, OR 0.44, 95% CI: 0.21 to 0.92, *p* = 0.029) and myocardial infarction (IVW, OR 0.39,95% CI: 0.15 to 0.98, *p* = 0.045) and a higher HDL-C (IVW, β 0.47, 95% CI: 0.24 to 0.70, *p* = 4×10^−5^).

**Discussion:**

The present findings suggest that raw vegetable intake is beneficial for weight loss and prevention of ASCVD.

## Graphical abstract

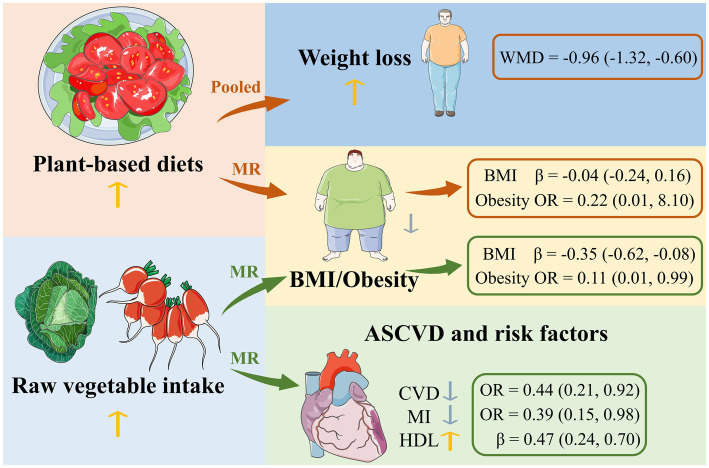

## Highlights

- A plant-based diet is strongly associated with weight loss.
- Raw vegetable intake has a negative causal effect on obesity.
- Raw vegetable intake favors the prevention of ASCVD.

## 1 Introduction

Obesity is an increasingly serious public health problem that leads to severe medical burdens for families and societies. It can also increase the risk of various chronic diseases such as type 2 diabetes, cardiovascular disease, etc. (1). Overweight and obesity are associated with more deaths worldwide than underweight (2). Obesity and its associated complications not only lead to increased morbidity and mortality, but also to a reduced quality of life (3). In almost all regions of the world, more people are overweight than underweight (2). The search is on for multimodal, interdisciplinary and individualized obesity therapies, including nutritional interventions or guidelines. Diet is a critical factor in the management of body weight and health worldwide (4). There is moderate evidence that dietary patterns emphasizing whole grains, vegetables, fruits, legumes and seafood are associated with lower body weight and obesity risk (5). Among the different dietary patterns, plant-based diets are increasingly being studied with indices such as the overall plant-based diet index (PDI), which offers more flexibility compared to a binary classification of vegetarianism and facilitates translation into dietary recommendations (4). Nowadays, there is an increasing trend to switch to a plant-based diet due to health benefits such as weight control. However, the relationship between plant-based diets and weight loss is not yet fully understood. Therefore, we conducted a pooled analysis of randomized clinical trials (6–29) and a Mendelian randomization (MR) analysis examining the effects of a plant-based diet on weight loss to clarify this association. Although there are several categories of plant-based diets (30), they can be divided into two main types: the lacto-ovo-vegetarian diet, which excludes meat but allows the consumption of eggs and milk, and the vegan diet, which rejects all animal products (31). In the pooled analysis, we use the two-category method for subgroup analysis.

A plant-based diet has been shown to be an effective means of improving plasma lipid profile and reducing the incidence of hypertension, ischemic heart disease, stroke, metabolic syndrome, and diabetes (32–36). Atherosclerotic cardiovascular disease (ASCVD) is defined as atherosclerotic vascular disease, which mainly includes coronary atherosclerotic heart disease, ischemic stroke, etc. It remains the leading cause of death worldwide, and a well-planned plant-based diet offers benefits in preventing and reversing atherosclerosis and reducing risk factors for ASCVD (7, 34, 37). Risk factors for ASCVD primarily include hyperlipidemia, hypertension, diabetes, obesity, etc. The American Heart Association/American College of Cardiology (AHA/ACC) has issued dietary recommendations, including the Dietary Approaches to Stop Hypertension (DASH) diet, the Mediterranean diet and the vegetarian diet, which help to fulfill the AHA/ACC guidelines. The recommendations emphasize increased consumption of vegetables, fruits, whole grains and legumes, but discourage red meat, sweets and sugar-sweetened beverages, as well as processed foods high in sugar, salt and fat or low in fiber (35). When it comes to the causal relationship between a plant-based diet and ASCVD, an MR study is urgently needed. The MR study considers genetic variants as exposure to determine whether an observed association between exposure and outcome is consistent with a causal effect (38, 39). It is based on Mendel’s second law (law of independent selection), which states that in the formation of germ cells, alleles are randomly segregated and non-alleles are freely combined. In this way, biases due to confounding and reverse causality can be effectively eliminated (38). To date, no MR study has investigated the causal relationship between plant-based diets and ASCVD. Therefore, we conducted a two-sample MR study following the pooled analysis described above.

## 2 Materials and methods

### 2.1 Pooled analysis

#### 2.1.1 Database and search strategy

We conducted this pooled analysis according to PRISMA guidelines and the Cochrane Handbook Version 6.3, 2022 (PROSPERO registration number: CRD42024498299). Based on the search strategy of the Cochrane Collaboration (40), we searched the PubMed and EMBASE databases (from inception to December 20, 2023) for randomized controlled trials on the effects of a plant-based diet on weight loss compared to a non-plant-based diet. The following keywords were used to search for relevant publications: (“weight”) AND (“vegan” OR “vegetarianism” OR “vegetarian” OR “plant-based diet”), without language restriction. Additional references were taken from the bibliographies of the identified studies.

#### 2.1.2 Study selection

Our analyses included randomized controlled trials that compared a vegan or lacto-ovo-vegetarian diet with a non-vegetarian diet, as measured by change in body weight. Studies were excluded if they were not from the original literature or if detailed information was not available. Studies that combined physical activity or other dietary interventions were also excluded, while studies that included psychological counseling or intervention were included.

#### 2.1.3 Data extraction and analysis

For each eligible study, information was extracted on the year of publication, population conditions, sample size, type of dietary intervention, follow-up periods, and changes in body weight. The means and standard deviations (SD) of weight changes were extracted or calculated. In the studies with repeated measures during the follow-up periods, data were extracted at the end of the intervention, with the exception of two studies, as in one study (19) the second 12 weeks of the diet were combined with aerobic exercise and in another study (23) participants were encouraged rather than forced to follow their assigned diet according to their own interests after a 2 months assessment. In a study (25) in which subjects’ dietary preferences were determined before they were randomly assigned to a lacto-ovo-vegetarian or a control diet, we separated the group with preferences from the group without preferences to allow comparison within each group. In addition, only percentages and not specific values were reported in this study, so we used the percentage data for the analysis.

Given the different population characteristics and sample sizes of the included studies, a random effects model was used (41). Weighted mean differences were calculated for identical outcome measures in the vegan/lacto-ovo-vegetarian and non-vegetarian diet groups. The I-squared statistic was used to examine the heterogeneity of treatment effects between the included studies. Due to the high values of the I-squared statistic, the random effects model we used was appropriate. Study characteristics that were potential sources of heterogeneity included the type of intervention diet (vegan vs. lacto-ovo-vegetarian), the inclusion of energy restriction in the intervention diet (no energy restriction, energy restriction for the control group only, energy restriction for both groups, and energy restriction during fasting), study duration (longer vs. less than 12 weeks), the gender involved in the studies (women >80% vs. others) and the population conditions (participants with a body mass index [BMI] ≥ 25, patients with other diseases and healthy subjects). Subgroup analyses were performed by pooling effect estimates based on the main areas of study-level sources of bias and significant factors mentioned above. In one study, the type of plant-based diet was changed (17). In the subgroup analyses, we categorized the study as lacto-ovo-vegetarian because the intervention duration was longer for lacto-ovo-vegetarian diets. Publication bias was analyzed by creating a funnel plot and the Egger test. All statistical procedures were carried out using Stata vIC15.0 software.

### 2.2 Mendelian randomization analysis

#### 2.2.1 Data sources

We considered a plant-based diet and raw vegetable intake as exposure. BMI, obesity, coronary heart disease, myocardial infarction, ischemic stroke, hypertension, type 2 diabetes, hyperlipidemia, systolic blood pressure, diastolic blood pressure, triglycerides, high-density lipoprotein (HDL) cholesterol, low-density lipoprotein (LDL) cholesterol, fasting blood glucose and HbA1c were considered as outcomes. The data sources for the exposures and outcomes considered in this study are all from the public database “IEU OPEN GWAS PROJECT” (https://gwas.mrcieu.ac.uk/, accessed December 30, 2023). More detailed information can be found in the Supplementary Table S1. Only data from European populations were used to avoid bias due to different populations.

#### 2.2.2 Selection of genetic instruments

We used genome-wide association studies (GWAS), which were mainly conducted in people of European descent, as a data source for the genetic variants of plant-based diets and raw vegetable intake. In this study, *p* < 5 × 10^−8^ was used as a threshold for screening single nucleotide polymorphisms (SNPs) strongly associated with exposure factors. If the number of SNPs was insufficient for MR analysis, the threshold was lowered stepwise to ensure that appropriate SNPs were included. First, a statistical approach was used to select SNPs that were associated with a plant-based diet (*p* < 5 × 10^−5^) and raw vegetable intake (*p* < 5 × 10^−8^) and were in low linkage disequilibrium with other SNPs ([LD] r^2^ < 0.001) within a 10,000 kb clump distance. Second, we excluded SNPs that were closely related to outcomes and were not available in the GWAS of outcomes. Third, palindromic and incompatible SNPs were also eliminated after harmonization. Finally, we calculated the F-statistic to quantify the strength of genetic variants and discarded SNPs with an F-statistic of less than 10.

#### 2.2.3 Statistical analysis

We used different MR approaches to estimate causality, namely the inverse-variance weighted (IVW), the weighted median and the MR-Egger method. The IVW random effects method was performed as the primary analysis, combining Wald ratio estimates to obtain a consistent estimate of the causal effect of exposure on outcome (42, 43). The weighted median assumes that at least 50% of the instruments are valid (44, 45). MR-Egger is the least informative method, but still provides consistent estimates considering pleiotropy when all instruments are invalid (45). A consistent effect across all MR approaches strengthens the causal evidence.

Sensitivity analyses were then performed to assess the robustness of the associations and possible pleiotropy. We used the Cochran Q test from the IVW approach to represent horizontal pleiotropy (46). The intercept obtained from MR-Egger regression was an indicator of directional pleiotropy (*p* < 0.05 was considered as the presence of pleiotropy) (47). A leave-one-out analysis was performed to assess whether the pooled IVW estimate was influenced by a single SNP (48).

All analyses were performed using R software (version 4.2.1) and the R package TwoSampleMR (version 0.5.7) (49, 50). Results were expressed as odds ratios (ORs) or β with the corresponding 95% confidence intervals (CIs). To control for false-positive results due to multiple testing, we used the Bonferroni correction adjusted for the number of primary exposures and outcomes in this study, and the *p* value <0.0016 (0.05/30) was considered statistically significant. *p* values between 0.0016 and 0.05 were considered to indicate a possible association.

## 3 Results

### 3.1 Pooled analysis

The literature search yielded 3,072 eligible studies. After excluding 217 duplicates and 2,751 articles based on the title and abstract, 107 were selected for full-text review (Supplementary Figure S1). We excluded 56 studies that were not clinical trials in adults, 8 studies where the main intervention did not include a vegan or lacto-ovo-vegetarian diet, 3 studies where additional interventions affected weight, 10 studies that were duplicates of results from the same research groups, 1 study where only the protocol but no outcomes were reported (not completed), and 5 studies where body weight was not reported, so we identified a total of 24 studies that met the inclusion criteria and were included in the final analysis.

#### 3.1.1 Study characteristics

**Figure 1:**
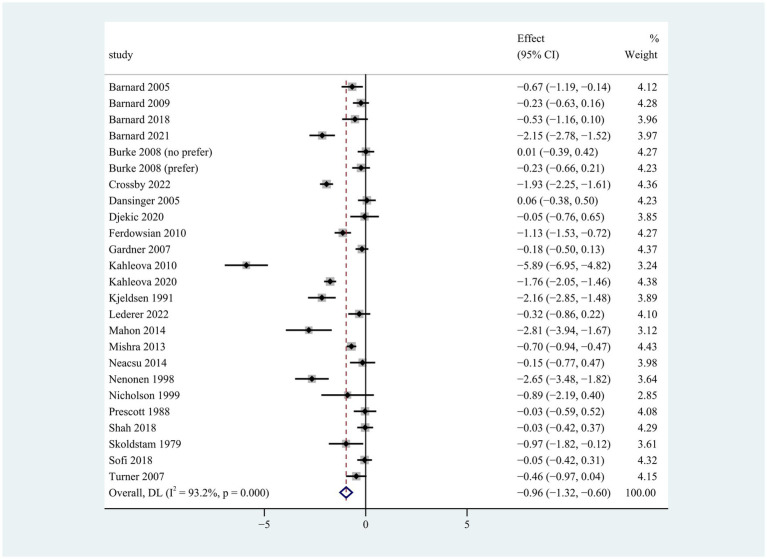
Pooled weighted mean differences in weight loss between plant-based and non-plant-based diets. The effects on the estimated weight loss for each study were presented as filled squares; the error bars indicate the 95% CIs. The pooled estimate of −0.96 kg (95% CI, −1.32 to −0.60) weight loss was presented as a diamond.

**Figure 2:**
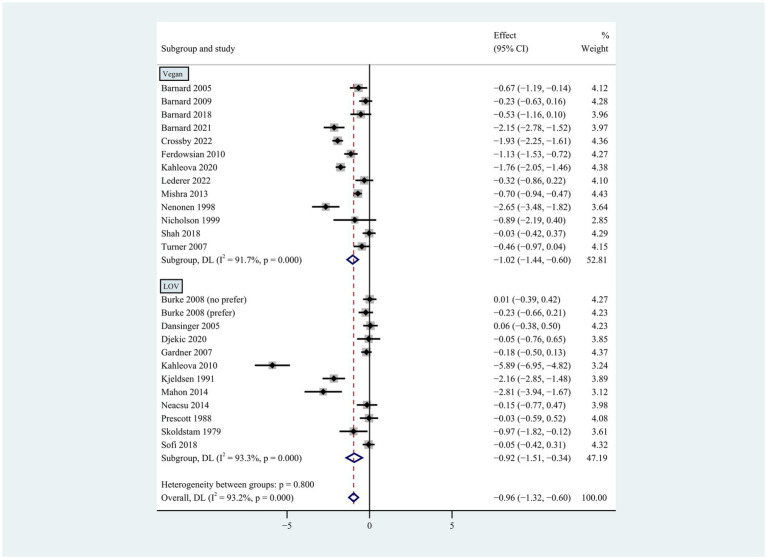
Pooled weighted mean differences in weight loss by dietary subgroups. Vegan diet vs. lacto-ovo-vegetarian (LOV) diet. Vegan diet was defined as abstaining from all animal products, while lacto-ovo-vegetarian diet avoided meat consumption but allowed milk and eggs. The effects on the estimated weight reduction for each study were presented as filled squares; the error bars indicate the 95% CIs. The pooled estimate of weight loss was presented as a diamond.

**Figure 3:**
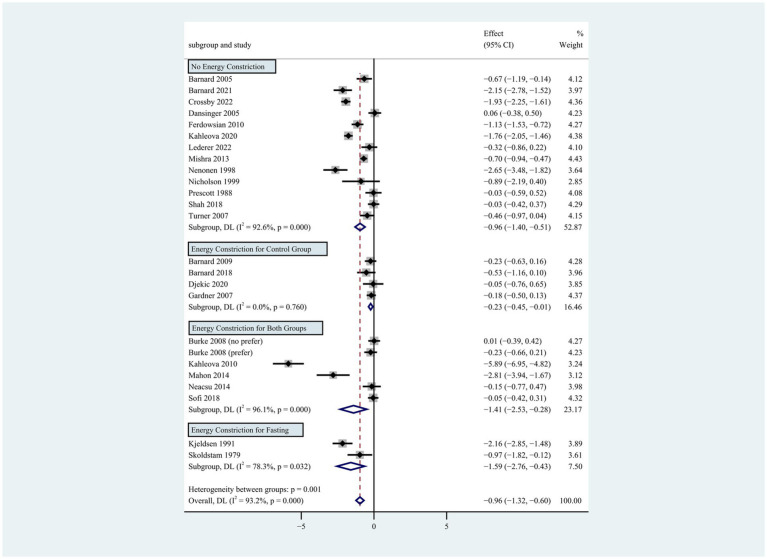
Pooled weighted mean differences in weight loss by subgroups with or without energy restriction. The effects on estimated weight loss for each trial were presented as filled squares; the error bars indicate the 95% CIs. The pooled estimate of weight loss was presented as a diamond.

**Figure 4:**
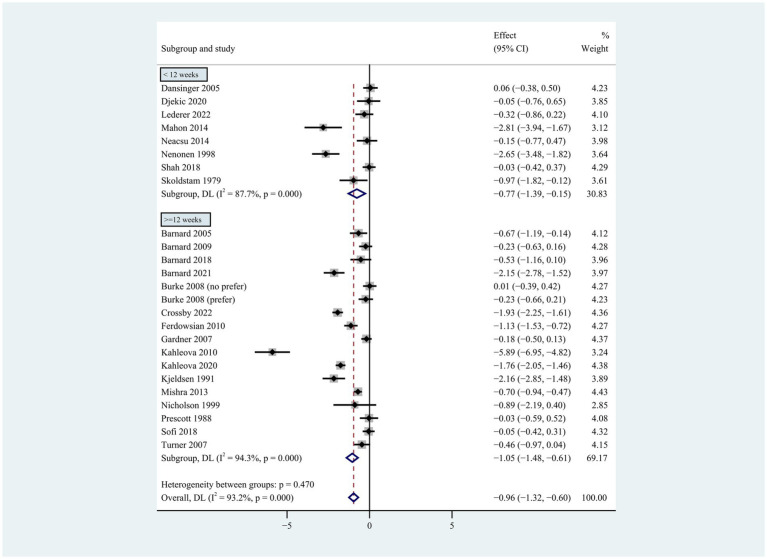
Pooled weighted mean differences in weight loss by subgrouping according to intervention duration. Intervention duration <12 weeks vs. ≥ 12 weeks. The effects on estimated weight loss for each trial were presented as filled squares; error bars indicate 95% CIs. The pooled estimate of weight loss was presented as a diamond.

**Figure 5:**
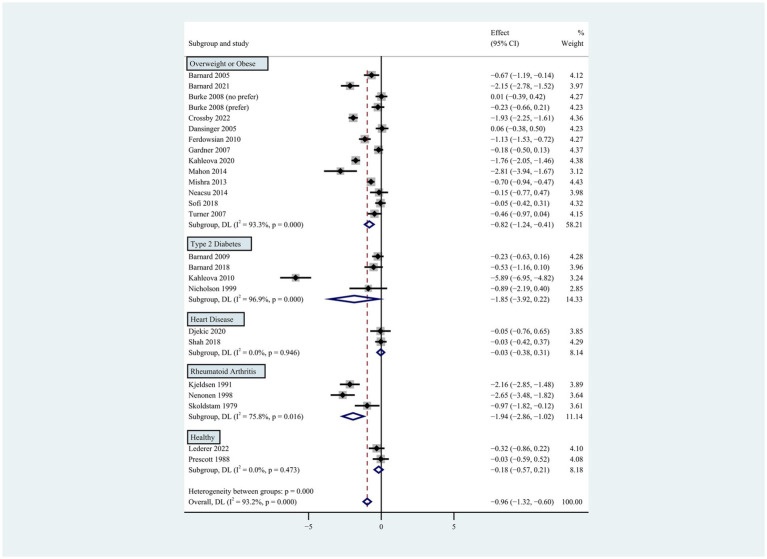
Pooled weighted mean differences in weight loss by subgroups, including overweight or obese population, type 2 diabetes, heart disease, rheumatoid arthritis and healthy population. The effects on estimated weight loss for each study were presented as filled squares; the error bars indicate the 95% CIs. The pooled estimate of weight loss was presented as a diamond.

**Figure 6:**
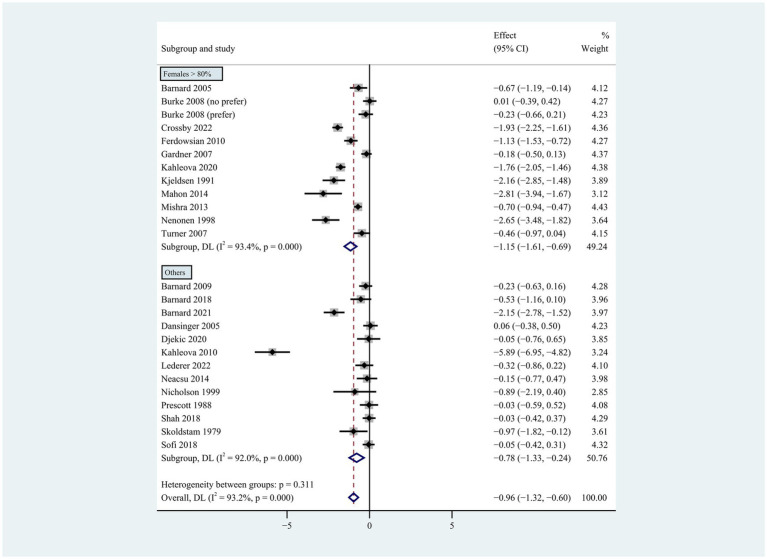
Pooled weighted mean differences in weight loss by gender subgroup. Gender composition female >80% vs. other. The effects on estimated weight loss for each trial were presented as filled squares; the error bars indicate the 95% CIs. The pooled estimate of weight loss was presented as a diamond.

Supplementary Table S2 summarizes the characteristics of the 24 eligible studies. A total of 2,223 subjects of both sexes with baseline ages ranging from 18 to 82 years were included in this analysis. Postmenopausal women were recruited in 3 studies. The proportion of male subjects ranged from 0 to 100%. Thirteen studies included overweight or obese patients, 4 studies included patients with type 2 diabetes, 3 studies included patients with rheumatoid arthritis and 2 studies included patients with heart disease. Vegan and lacto-ovo-vegetarian diets were selected as intervention diets in 13 and 11 studies, respectively. Of the 13 studies on vegan diets, 10 used low-fat (≤ 10%) recipes. The design of the non-vegetarian diets varied from study to study and included the National Cholesterol Education Program (NCEP) diet, low-fat diets, anti-diabetes diets, weight-loss diets, Mediterranean diets, high-protein, low-calorie diets, and habitual diet recipes. Of the 11 studies that applied energy restriction, 5 applied the restriction to both the intervention and control groups, while 1 study applied a 1 week fasting energy restriction to the intervention group only (8). Of the remaining 13 studies without energy restriction in the intervention groups, 1 study applied energy restriction in its overweight control group. The intervention periods in these studies ranged from 2 weeks to 2 years. In the subgroup analyses, population status (*p* < 0.001) and energy restriction (*p* = 0.001) were identified as the study-level factors that significantly influenced the pooling results (Figures 1–6).

#### 3.1.2 Weight change

Compared to the control group, participants on the plant-based diet lost more weight during the intervention period of 2 to 96 weeks (weighted mean difference [WMD]: −0.96 kg; 95% CI: −1.32 to −0.60) (Figure 1). There was apparent heterogeneity in weight change (*p* < 0.001 for heterogeneity, I^2^ = 93.2%), so subgroup analyses were conducted for type of plant-based diet, intervention duration, population characteristics, energy restriction, and gender.

Individuals who followed a vegan diet (13 studies with a duration of 8 to 96 weeks) lost slightly more body weight than those who followed a lacto-ovo-vegetarian diet (11 studies with a duration of 2 to 72 weeks) compared to those who followed a control diet. However, the result of the heterogeneity test between the two subgroups was not statistically significant (heterogeneity between two subgroups: *p* = 0.800). Specifically, the weighted mean weight reduction in subjects assigned to a vegan diet was −1.02 kg (95% CI: −1.44 to −0.60, *p* < 0.001 for heterogeneity, I^2^ = 91.7%), while the corresponding weight change in lacto-ovo-vegetarian diets was −0.92 kg (95% CI: −1.51 to −0.34, *p* < 0.001 for heterogeneity, I^2^ = 93.2%) (Figure 2). Eleven studies developed energy-restricted diets (including energy restriction for the control group only, energy restriction for both groups, and energy restriction during fasting) in intervention periods ranging from 2 to 74 weeks. Studies with energy restriction for the control group only showed the least weight loss (−0.23 kg; 95% CI: −0.45 to −0.01, *p* = 0.760 for heterogeneity, I^2^ = 0.0%), while studies with energy restriction during fasting showed the greatest weight loss (−1.59 kg; 95% CI: −2.76 to −0.43, *p* < 0.032 for heterogeneity, I^2^ = 78.3%). The weighted mean weight loss in the studies without energy restriction was −0.96 kg (95% CI: −1.40 to −0.51, *p* < 0.001 for heterogeneity, I^2^ = 92.6%) for interventions of 4 to 96 weeks, and the result was similar to the analysis of all studies. The weighted mean weight loss in the energy-restricted trials for both groups was −1.41 kg (95% CI: −2.53 to −0.28, *p* < 0.001 for heterogeneity, I^2^ = 96.1%) (Figure 3). The weight loss achieved in 8 studies with a duration of less than 12 weeks (2 ~ 12 weeks) was lower (−0.77 kg; 95% CI: −1.39 to −0.15, *p* < 0.001 for heterogeneity, I^2^ = 87.7%) than in the 13 studies with an intervention duration of more than 12 weeks (12 ~ 96 weeks) (−1.05 kg; 95% CI: −1.48 to −0.61, *p* < 0.001 for heterogeneity, I^2^ = 94.3%) (Figure 4). In 4 studies of patients with type 2 diabetes (−1.85 kg; 95% CI: −3.92 to 0.22, *p* < 0.001 for heterogeneity, I^2^ = 96.9%) and 3 studies of patients with rheumatoid arthritis (−1.94 kg; 95% CI: −2.86 to −1.02, *p* = 0.016 for heterogeneity, I^2^ = 75.8%), a plant-based diet resulted in greater weight loss in each case. However, 2 studies limited to heart disease (−0.03 kg; 95% CI: −0.38 to 0.31, *p* = 0.946 for heterogeneity, I^2^ = 0.0%) and 2 studies limited to healthy individuals (−0.18 kg; 95% CI: −0.57 to 0.21, *p* = 0.473 for heterogeneity, I^2^ = 0.0%) resulted in almost no weight loss with the interventions. Thirteen studies that included subjects who were overweight or obese at baseline showed similar weight loss (−0.82 kg; 95% CI: −1.24 to −0.41, *p* < 0.001 for heterogeneity, I^2^ = 93.3%) to the analysis of all studies (Figure 5). Gender was also used as a factor for subgroup analyses. Eleven studies that were limited to women >80% had greater weight loss (−1.15 kg; 95% CI: −1.61 to −0.69, *p* < 0.001 for heterogeneity, I^2^ = 93.4%) than the other studies (−0.78 kg; 95% CI: −1.33 to −0.24, *p* < 0.001 for heterogeneity, I^2^ = 92.0%) (Figure 6).

A sensitivity analysis was also performed by removing one study and estimating whether the results of the remaining studies would be significantly affected by a single study. The result showed that the conclusion from this pooled analysis was relatively robust and conceivable (Supplementary Figure S2).

#### 3.1.3 Publication bias

The funnel plot (Supplementary Figure S3A) showed an asymmetry that was probably influenced by the number of included studies. Nevertheless, there was no indication of a significant publication bias (Egger test *p* = 0.274) (Supplementary Figure S3B).

### 3.2 Mendelian randomization analysis

After removing SNPs that were not strongly relevant to exposures and exhibited the linkage disequilibrium phenomenon, SNPs that met the conditions were selected for analysis. Detailed information on these SNPs can be found in the Supplementary Tables S3, S4. All of these SNPs showed strong associations with the exposures (F-statistic >10) and were not directly associated with the outcomes. Supplementary Figure S4 shows the entire MR analysis procedure.

#### 3.2.1 Causal effects of a plant-based diet/raw vegetable intake on obesity or BMI

When analyzing the causal effects of a plant-based diet, we performed a rigorous screening of SNPs and finally included 13 SNPs for the analysis of the obesity outcome and 17 for BMI. A plant-based diet was not significantly associated with a lower risk of obesity (IVW, OR = 0.22, 95% CI: 0.01 to 8.10, *p* = 0.411) (Supplementary Figure S5) and lower BMI (IVW, β = −0.04, 95% CI: −0.24 to 0.16, *p* = 0.678) (Supplementary Figure S6).

**Figure 7:**
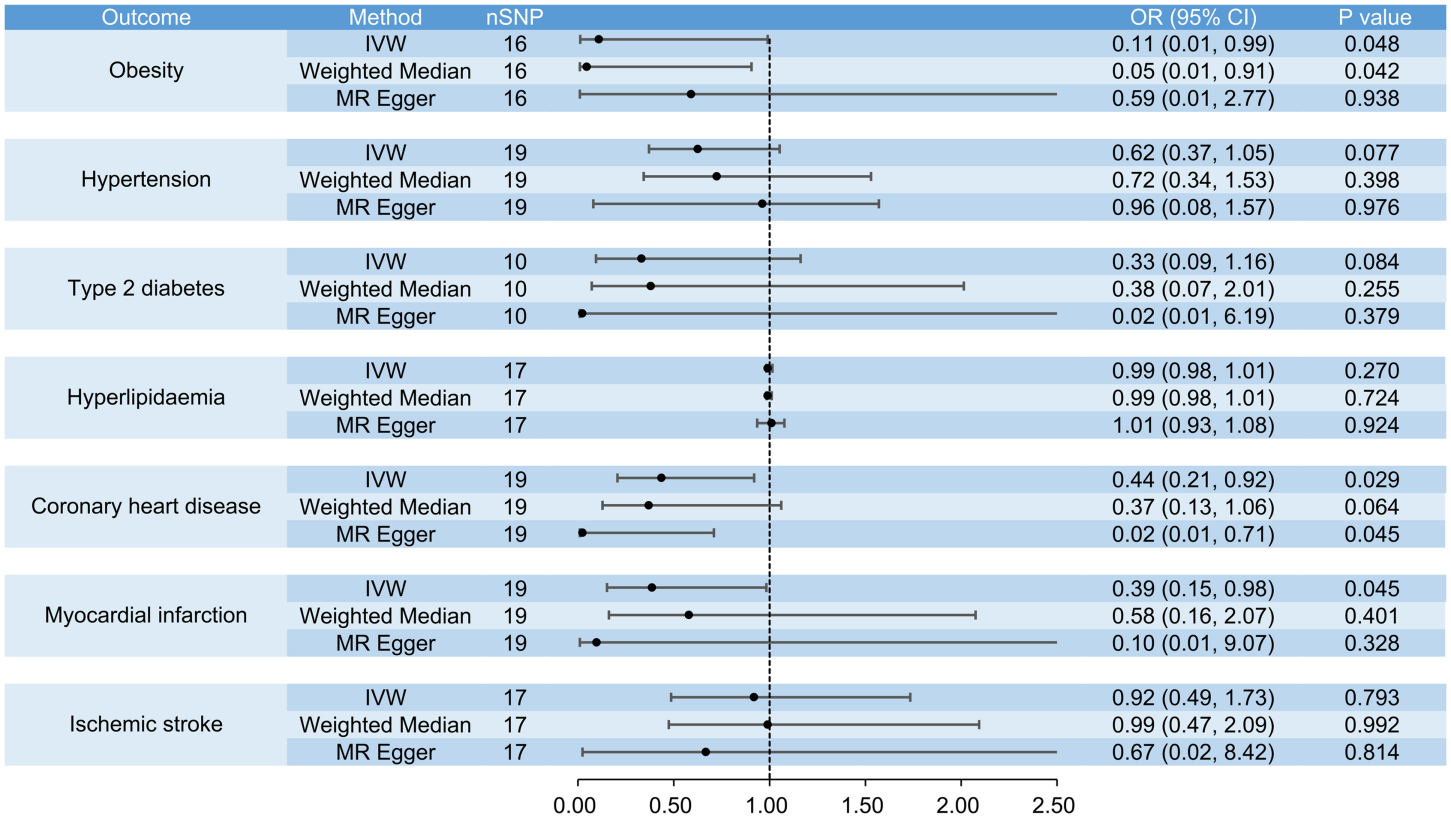
The causal effects of raw vegetable intake on obesity, hypertension, type 2 diabetes, hyperlipidemia, coronary heart disease, myocardial infarction and ischemic stroke.

**Figure 8:**
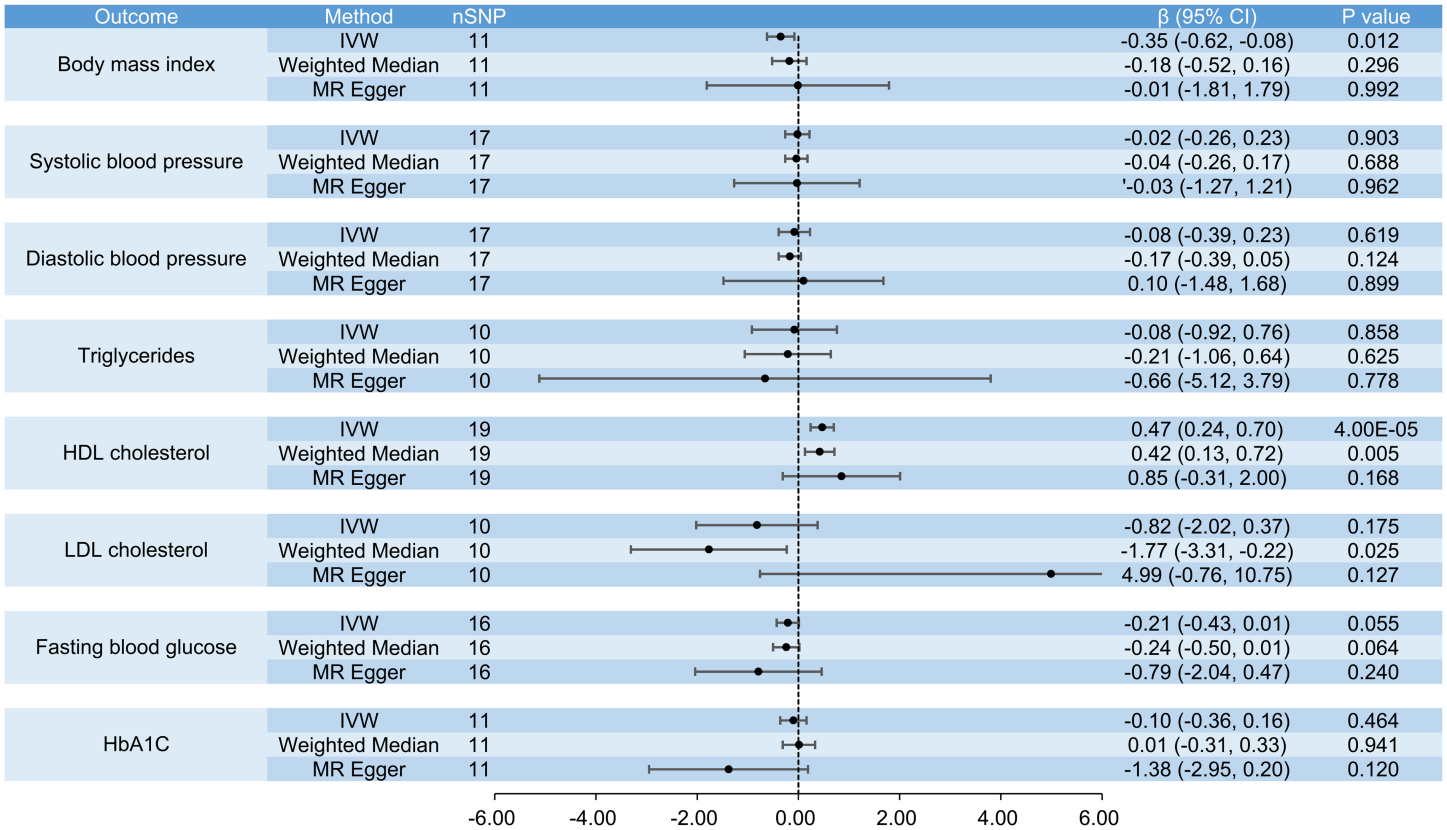
The causal effects of raw vegetable intake on BMI, systolic blood pressure, diastolic blood pressure, triglycerides, HDL cholesterol, LDL cholesterol, fasting blood glucose and HbA1c.

When analyzing the causal effects of raw vegetable intake, we included 16 SNPs for the analysis of obesity and 11 for BMI. Genetically increased consumption of raw vegetables was potentially associated with a lower risk of obesity when using the IVW (OR = 0.11, 95% CI: 0.01 to 0.99, *p* = 0.048) and weighted median methods (OR = 0.05, 95% CI: 0.01 to 0.91, *p* = 0.042), but the result of the MR-Egger regression method was not statistically significant (OR = 0.59, 95% CI: 0.01 to 2.77, *p* = 0.938) (Figure 7). Genetically increased consumption of raw vegetables was also potentially associated with lower BMI using the IVW method (β = −0.35, 95% CI: −0.62 to −0.08, *p* = 0.012), although the results of the MR-Egger regression method (β = −0.01, 95% CI: −1.81 to 1.79, *p* = 0.992) and the weighted median method (β = −0.18, 95% CI: −0.52 to 0.16, *p* = 0.296) were not statistically significant (Figure 8).

#### 3.2.2 Causal effects of a plant-based diet/raw vegetable intake on ASCVD and risk factors

In analyzing the causal effects of a plant-based diet, we performed a rigorous screening of SNPs and identified 37 SNPs for the analyses of coronary heart disease, ischemic stroke, hypertension, type 2 diabetes, hyperlipidemia and systolic blood pressure, 35 SNPs for the analyses of myocardial infarction, diastolic blood pressure, triglycerides and HDL cholesterol, 18 SNPs for LDL cholesterol, 14 SNPs for fasting blood glucose, and 32 SNPs for HbA1c. There was no evidence that a plant-based diet was causally associated with coronary heart disease (IVW, OR = 0.98, 95% CI: 0.95 to 1.01, *p* = 0.189), myocardial infarction (IVW, OR = 0.72, 95% CI: 0.21 to 2.49, *p* = 0.609), ischemic stroke (IVW, OR = 0.97, 95% CI: 0.46 to 2.03, *p* = 0.935), hypertension (IVW, OR = 0.99, 95% CI: 0.98 to 1.01, *p* = 0.889), type 2 diabetes (IVW, OR = 0.99, 95% CI: 0.98 to 1.01, *p* = 0.231), hyperlipidemia (IVW, OR = 1.01, 95% CI: 0.99 to 1.02, *p* = 0.835), systolic blood pressure (IVW, β = −0.07, 95% CI: −0.30 to 0.15, *p* = 0.518), diastolic blood pressure (IVW, β = −0.05, 95% CI: −2.65 to 2.55, *p* = 0.969), triglycerides (IVW, β = −0.01, 95% CI: −0.24 to 0.23, *p* = 0.961), HDL cholesterol (IVW, β = 0.11, 95% CI: −0.14 to 0.37, *p* = 0.370), LDL cholesterol (IVW, β = −0.05, 95% CI: −0.64 to 0.54, *p* = 0.872), fasting blood glucose (IVW, β = −0.08, 95% CI: −0.57 to 0.41, *p* = 0.757) and HbA1c (IVW, β = −0.04, 95% CI: −0.56 to 0.48, *p* = 0.875) (Supplementary Figures S5, S6).

In the analyses of the causal effects of raw vegetable intake, we included 19 SNPs for the analyses of coronary heart disease, myocardial infarction, hypertension and HDL cholesterol, 17 SNPs for the analyses of ischemic stroke, hyperlipidemia, systolic and diastolic blood pressure, 10 SNPs for the analyses of type 2 diabetes, triglycerides and LDL cholesterol, 16 SNPs for fasting blood glucose, and 11 SNPs for HbA1c. Genetically increased consumption of raw vegetables was significantly associated with higher HDL cholesterol when the IVW method was used (β = 0.47, 95% CI: 0.24 to 0.70, *p* = 4 × 10^−5^) and was also potentially associated with higher HDL cholesterol when the weighted median method was used (β = 0.42, 95% CI: 0.13 to 0.72, *p* = 0.005). A similar result was obtained using the MR-Egger regression method (β = 0.85, 95% CI: −0.31 to 2.00, *p* = 0.168), although the result was not statistically significant. It was also potentially associated with a lower risk of coronary heart disease (IVW, OR = 0.44, 95% CI: 0.21 to 0.92, *p* = 0.029; MR-Egger, OR = 0.02, 95% CI: 0.01 to 0.71, *p* = 0.045) and myocardial infarction (IVW, OR = 0.39, 95% CI: 0.15 to 0.98, *p* = 0.045), but not with ischemic stroke (IVW, OR = 0.92, 95% CI: 0.49 to 1.73, *p* = 0.793), hypertension (IVW, OR = 0.62, 95% CI: 0.37 to 1.05, *p* = 0.077), type 2 diabetes (IVW, OR = 0.33, 95% CI: 0.09 to 1.16, *p* = 0.084), hyperlipidemia (IVW, OR = 0.99, 95% CI: 0.98 to 1.01, *p* = 0.270), systolic blood pressure (IVW, β = −0.02, 95% CI: −0.26 to 0.23, *p* = 0.903), diastolic blood pressure (IVW, β = −0.08, 95% CI: −0.39 to 0.23, *p* = 0.619), triglycerides (IVW, β = −0.08, 95% CI: −0.92 to 0.76, *p* = 0.858), LDL cholesterol (IVW, β = −0.82, 95% CI: −2.02 to 0.37, *p* = 0.175), fasting blood glucose (IVW, β = −0.21, 95% CI: −0.43 to 0.01, *p* = 0.055) and HbA1c (IVW, β = −0.10, 95% CI: −0.36 to 0.16, *p* = 0.464) (Figures 7, 8).

#### 3.2.3 Robustness of the positive results

After obtaining the above positive results, we performed further sensitivity analyses. The *p* values of the Egger intercept were greater than 0.05, indicating that there was no directional pleiotropy in the above analyses (Supplementary Table S5). The *p* values of the Cochran Q-test were also greater than 0.05, indicating that there was no strong unbalanced horizontal pleiotropy (Supplementary Table S6). Leave-one-out analyses showed that causality remained unchanged after elimination of a single SNP (Supplementary Figures S7A–S11A). Scatter plots visually depicted the causal relationship (Supplementary Figures S7B–S11B). Diagnostic MR analyses with funnel plots were also performed (Supplementary Figures S7C–S11C). Thus, all positive results were robust and not dramatically affected by bias.

## 4 Discussion

In the pooled analysis of the randomized controlled trials comparing the changes in body weight between the subjects who ate a plant-based diet and those who did not, the former showed a reduction in body weight of about 1 kg compared to the latter. However, the result was not consistent with the previous pooled analysis (31), possibly due to differences in the included studies. In subjects on a plant-based diet, a vegan diet resulted in slightly greater weight loss than a lacto-ovo-vegetarian diet. Weight loss was also significantly greater in studies with energy restriction for both groups or with fasting. The results also suggest that the effect of the intervention appears to increase over time. Gender composition also appears to contribute to the differences in weight loss, but there was a lack of data from studies in which men outnumbered women, so it was difficult to draw conclusions. Unfortunately, there was no evidence in the MR analysis that a plant-based diet was causally related to the above outcomes. Nevertheless, we note that increased consumption of raw vegetables was potentially associated with lower BMI and lower risk of obesity. It was also potentially associated with a lower risk of coronary heart disease and myocardial infarction and was significantly associated with higher HDL cholesterol, but not with ischemic stroke, hypertension, type 2 diabetes, hyperlipidemia, systolic blood pressure, diastolic blood pressure, triglycerides, LDL cholesterol, fasting blood glucose, and HbA1c.

Data from the Adventist Health Study (AHS) have shown that BMI increases with the proportion of animal foods in the diet (51). According to the results of the EPIC-Oxford study (52), there was hardly any difference in weight gain between meat eaters, fish eaters, vegetarians and vegans. In the past, plant extracts such as vegetables and fruits have been found to regulate adipogenesis and attenuate obesity (53), and the use of a plant-based diet has been investigated as a potential treatment for obesity. Therefore, a plant-based diet is a viable option for people who want to control their body weight and improve the quality of their diet to prevent and treat metabolic diseases (54). In addition, vegetarians have a relatively low risk of diverticular disease, kidney stones, cataracts, dementia and some cancers (52). According to the results of our literature search, the last pooled analysis on the association between plant-based diets and weight loss was published in 2015, 8 years ago (31). Since then, numerous randomized controlled trials and observational studies have provided data on weight loss in individuals on a plant-based diet, which should be pooled for an updated analysis. Therefore, we selected the association between plant-based diets and weight loss as the subject of our pooled analysis and also performed an MR analysis to assess the causal relationship between the two. In recent years, several studies have shown that a plant-based diet is associated with a lower risk of ASCVD mortality and morbidity. Kahleova H et al. concluded that a plant-based diet is the only dietary pattern that has been shown to reverse coronary heart disease, as it can prevent and reverse atherosclerosis. They also show effects on the prevention and treatment of cerebrovascular disease. A plant-based diet is also associated with lower blood lipid levels and lower blood pressure and may reduce the risk of type 2 diabetes (7, 34). Dinu M et al. concluded that the risk of ischemic heart disease incidence and/or mortality is lower in vegetarians and vegans compared to omnivores (RR 0.75, 95% CI: 0.68 to 0.82) (32). Fraser GE et al. suggested that the lifetime risk of ischemic heart disease is reduced by 37% in male vegetarians compared to non-vegetarians (33). There has been no MR analysis of vegetarianism and ASCVD, apart from one MR study that concluded that cheese consumption is inversely associated with coronary atherosclerosis and arterial stiffness (55). Although cheese is a dairy product, it cannot fully represent the vegetarian diet. It is therefore necessary to investigate the causality between the two.

The results of the MR analysis are not fully consistent with the results of the pooled analysis, which can be explained by the following reasons. First, although sensitivity analysis and publication bias analysis were conducted to confirm the reliability, the heterogeneity of the pooled analysis was unavoidably high, so the analysis results may not fully reflect the actual situation. Second, there are few GWAS on plant-based diets, so the results we obtained from the MR analysis may not be representative enough. Therefore, it is important that we evaluate more GWAS to perform a more reliable analysis. Nevertheless, we prefer the conclusions from the MR analysis because it is inherently better and can effectively avoid bias from confounding factors and reverse causality. We hypothesize that the relationship between plant-based diets and weight loss is not causal, but only correlative. Since increased vegetable consumption was causally associated with weight loss in the MR analysis, the composition of the vegetarian diet in the studies included in the pooled analysis was predominantly vegetables, which explains why the results of the pooled analysis contradict those of the MR analysis.

There are several reasons why higher vegetable consumption may be associated with weight loss. First, vegetables generally have a low energy density (56), so higher vegetable intake reduces energy intake to some extent, regardless of the intake of specific foods. When energy intake is lower than the body’s energy needs, the body utilizes stored fat reserves, leading to weight loss. Second, plant foods are rich in dietary fiber, which can hinder the absorption of food and act as a satiety factor that induces a feeling of fullness and reduces food intake, which has some effect on weight control (57). Dietary fiber can also promote intestinal peristalsis and accelerate the elimination of intestinal contents. In addition, a low intake of saturated fat and a high intake of various phytochemicals are likely to lead to a lower BMI (58). Third, consumption of high-fiber foods increases the number of chewing processes, which may slow eating and nutrient absorption in the small intestine (59). We also summarized the following mechanisms to explain raw vegetable consumption as a cardiovascular protective factor (34). First, raw vegetables contain phytosterols and unsaturated fats that lower blood cholesterol concentrations, and it also contains various substances (e.g., tocopherols, ascorbate, carotenoids, saponins, and flavonoids) that can reduce inflammation and oxidative stress, thereby reducing the risk of cardiovascular disease (60–62). Second, since insulin resistance and impaired insulin secretion are important mechanisms underlying cardiovascular disease morbidity and mortality (63, 64), raw vegetables may increase insulin sensitivity and stimulate insulin secretion (19). Third, raw vegetables are low in energy density, which also has benefits for cardiovascular health and longevity (65, 66).

Some limitations of this study should be considered. In the pooled analysis, the number of studies included in the subgroups was too small, so the effect needs to be confirmed by further studies. In addition, the plant-based diets had several specific characteristics, most of which were low in fat, but also low in calories, low in protein, and even gluten-free and uncooked. Each of these different types of plant-based diets should be investigated further. In MR analysis, our dietary habits are essentially a subjective behavior rather than an objective indicator, and the influence of genetic determinants could be attenuated or buffered by compensatory developmental processes, so considering genetic variants as exposure is likely to introduce bias. In addition, the current study is based on pooled data, and the sex-specific subgroup analyses were incomplete. Therefore, the gender-specific effects of exposures need to be further investigated.

## 5 Conclusion

A plant-based diet is closely linked to weight loss. However, the causal relationship between the two is still unclear and more in-depth studies are needed. To some extent, increased consumption of raw vegetables is beneficial for weight loss and the prevention of ASCVD.

## Data Availability

The original contributions presented in the study are included in the article/Supplementary material, further inquiries can be directed to the corresponding author.

## Supplementary material

SUPPLEMENTARY FIGURE S1 Flowchart of the pooled analysis.

SUPPLEMENTARY FIGURE S2 Result of the sensitive analysis. The result shows that the conclusion from this pooled analysis is robust and conceivable.

SUPPLEMENTARY FIGURE S3 Result of the publication bias analysis. **(A)** Funnel plot for visual and quantitative assessment of publication bias. The result shows an asymmetry in both the small and large sample groups, which was probably influenced by the number of included studies. **(B)** Egger’s test for quantitative assessment of publication bias. No evidence of significant publication bias (Egger’s test *p* = 0.274).

SUPPLEMENTARY FIGURE S4 Flowchart of the MR analysis.

SUPPLEMENTARY FIGURE S5 The causal effects of a plant-based diet on obesity, hypertension, type 2 diabetes, hyperlipidemia, coronary heart disease, myocardial infarction and ischemic stroke.

SUPPLEMENTARY FIGURE S6 The causal effects of a plant-based diet on BMI, systolic blood pressure, diastolic blood pressure, triglycerides, HDL cholesterol, LDL cholesterol, fasting blood glucose and HbA1c.

SUPPLEMENTARY FIGURE S7 The distribution of SNPs and sensitivity analyses for the causal effect of raw vegetable intake on BMI. **(A)** Forest plot of the leaveone-out sensitivity analysis. **(B)** Scatter plot for the causal effect. **(C)** The funnel plot for the distribution of SNPs.

SUPPLEMENTARY FIGURE S8 The distribution of SNPs and sensitivity analyses for the causal effect of raw vegetable intake on obesity. **(A)** Forest plot of the leaveone-out sensitivity analysis. **(B)** Scatter plot for the causal effect. **(C)** The funnel plot for the distribution of SNPs.

SUPPLEMENTARY FIGURE S9 The distribution of SNPs and sensitivity analyses for the causal effect of raw vegetable intake on coronary heart disease. **(A)** Forest plot of the leave-one-out sensitivity analysis. **(B)** Scatter plot for the causal effect. **(C)** The funnel plot for the distribution of SNPs.

SUPPLEMENTARY FIGURE S10 The distribution of SNPs and sensitivity analyses for the causal effect of raw vegetable intake on myocardial infarction. **(A)** Forest plot of the leave-one-out sensitivity analysis. **(B)** Scatter plot for the causal effect. **(C)** The funnel plot for the distribution of SNPs.

SUPPLEMENTARY FIGURE S11 The distribution of SNPs and sensitivity analyses for the causal effect of raw vegetable intake on HDL cholesterol. **(A)** Forest plot of the leave-one-out sensitivity analysis. **(B)** Scatter plot for the causal effect. **(C)** The funnel plot for the distribution of SNPs.

SUPPLEMENTARY TABLE S1 Details of the GWAS database.

SUPPLEMENTARY TABLE S2 General characteristics of the included studies for the pooled analysis.

SUPPLEMENTARY TABLE S3 Details of SNPs included in the analyses when plant-based diet was considered as an exposure.

SUPPLEMENTARY TABLE S4 Details of SNPs included in the analyses when raw vegetable intake was considered an exposure.

SUPPLEMENTARY TABLE S5 Tests for directional pleiotropy.

SUPPLEMENTARY TABLE S6 Tests for horizontal pleiotropy.
