# Supplementary material for: Relationship of vegetarianism with body weight loss and ASCVD
Source: Front Nutr. 2024 Aug 27;11:1419743. doi: 10.3389/fnut.2024.1419743 (PMC11389726; doi:10.3389/fnut.2024.1419743)
Supplement: Supplementary file 1 [file Data_Sheet_1.zip › Supplementary Figure Legends.docx]

**Supplementary Figure Legends**

**Supplementary Fig. S1.** Flowchart of the pooled analysis.

**Supplementary Fig. S2.** Result of the sensitive analysis. The result shows that the conclusion from this pooled analysis is robust and conceivable.

**Supplementary Fig. S3.** Result of the publication bias analysis. (A) Funnel plot for visual and quantitative assessment of publication bias. The result shows an asymmetry in both the small and large sample groups, which was probably influenced by the number of included studies. (B) Egger’s test for quantitative assessment of publication bias. No evidence of significant publication bias (Egger’s test P = 0.274).

**Supplementary Fig. S4.** Flowchart of the MR analysis.

**Supplementary Fig. S5.** The causal effects of a plant-based diet on obesity, hypertension, type 2 diabetes, hyperlipidemia, coronary heart disease, myocardial infarction and ischemic stroke.

**Supplementary Fig. S6.** The causal effects of a plant-based diet on BMI, systolic blood pressure, diastolic blood pressure, triglycerides, HDL cholesterol, LDL cholesterol, fasting blood glucose and HbA1c.

**Supplementary Fig. S7.** The distribution of SNPs and sensitivity analyses for the causal effect of raw vegetable intake on BMI. (A) Forest plot of the leave-one-out sensitivity analysis. (B) Scatter plot for the causal effect. (C) The funnel plot for the distribution of SNPs.

**Supplementary Fig. S8.** The distribution of SNPs and sensitivity analyses for the causal effect of raw vegetable intake on obesity. (A) Forest plot of the leave-one-out sensitivity analysis. (B) Scatter plot for the causal effect. (C) The funnel plot for the distribution of SNPs.

**Supplementary Fig. S9.** The distribution of SNPs and sensitivity analyses for the causal effect of raw vegetable intake on coronary heart disease. (A) Forest plot of the leave-one-out sensitivity analysis. (B) Scatter plot for the causal effect. (C) The funnel plot for the distribution of SNPs.

**Supplementary Fig. S10.** The distribution of SNPs and sensitivity analyses for the causal effect of raw vegetable intake on myocardial infarction. (A) Forest plot of the leave-one-out sensitivity analysis. (B) Scatter plot for the causal effect. (C) The funnel plot for the distribution of SNPs.

**Supplementary Fig. S11.** The distribution of SNPs and sensitivity analyses for the causal effect of raw vegetable intake on HDL cholesterol. (A) Forest plot of the leave-one-out sensitivity analysis. (B) Scatter plot for the causal effect. (C) The funnel plot for the distribution of SNPs.
