# Supplementary material for: Relationship of vegetarianism with body weight loss and ASCVD
Source: Front Nutr. 2024 Aug 27;11:1419743. doi: 10.3389/fnut.2024.1419743 (PMC11389726; doi:10.3389/fnut.2024.1419743)
Supplement: Supplementary file 1 [file Data_Sheet_1.zip › Table S1.DOCX]

| **GWAS ID** | **Year** | **Trait** | **Number of cases** | **Number of controls** | **Sample size** | **Ancestry** | **Sex** | **Pubmed ID** | **Web Source** |
| --- | --- | --- | --- | --- | --- | --- | --- | --- | --- |
| ukb-b-11679 | 2018 | Vegetarian | 2202 | 62747 | 64949 | European | Males and Females | NA | https://gwas.mrcieu.ac.uk/datasets/ukb-b-11679/ |
| ukb-b-1996 | 2018 | Raw vegetable intake | NA | NA | 435435 | European | Males and Females | NA | https://gwas.mrcieu.ac.uk/datasets/ukb-b-1996/ |
| ebi-a-GCST002783 | 2015 | Body mass index | NA | NA | 236781 | European | NA | 25673413 | https://gwas.mrcieu.ac.uk/datasets/ebi-a-GCST002783/ |
| ieu-b-40 | 2018 | Body mass index | NA | NA | 681275 | European | Males and Females | 30124842 | https://gwas.mrcieu.ac.uk/datasets/ieu-b-40/ |
| ieu-a-92 | 2013 | Obesity | 2896 | 47468 | 50364 | European | Males and Females | 23563607 | https://gwas.mrcieu.ac.uk/datasets/ieu-a-92/ |
| ebi-a-GCST001475 | 2012 | Obesity | 5530 | 8318 | 13848 | European | NA | 22484627 | https://gwas.mrcieu.ac.uk/datasets/ebi-a-GCST001475/ |
| finn-b-I9_CHD | 2021 | Coronary heart disease | 21012 | 197780 | 218792 | European | Males and Females | NA | https://gwas.mrcieu.ac.uk/datasets/finn-b-I9_CHD/ |
| ukb-b-1668 | 2018 | Atherosclerotic heart disease | 12171 | 450839 | 463010 | European | Males and Females | NA | https://gwas.mrcieu.ac.uk/datasets/ukb-b-1668/ |
| finn-b-I9_MI | 2021 | Myocardial infarction | 12801 | 187840 | 200641 | European | Males and Females | NA | https://gwas.mrcieu.ac.uk/datasets/finn-b-I9_MI/ |
| finn-b-I9_HYPTENSESS_EXNONE | 2021 | Hypertension | 42857 | 175935 | 218792 | European | Males and Females | NA | https://gwas.mrcieu.ac.uk/datasets/finn-b-I9_HYPTENSESS_EXNONE/ |
| ukb-d-I9_HYPTENS | 2018 | Hypertension | 1237 | 359957 | 361194 | European | Males and Females | NA | https://gwas.mrcieu.ac.uk/datasets/ukb-d-I9_HYPTENS/ |
| ieu-a-26 | 2012 | Type 2 diabetes | 12171 | 56862 | 69033 | European | Males and Females | 22885922 | https://gwas.mrcieu.ac.uk/datasets/ieu-a-26/ |
| ukb-b-13806 | 2018 | Type 2 diabetes | 2972 | 459961 | 462933 | European | Males and Females | NA | https://gwas.mrcieu.ac.uk/datasets/ukb-b-13806/ |
| ukb-b-17462 | 2018 | Hyperlipidaemia | 3439 | 459571 | 463010 | European | Males and Females | NA | https://gwas.mrcieu.ac.uk/datasets/ukb-b-17462/ |
| ebi-a-GCST005843 | 2018 | Ischemic stroke | 34217 | 406111 | 440328 | European | NA | 29531354 | https://gwas.mrcieu.ac.uk/datasets/ebi-a-GCST005843/ |
| ukb-a-360 | 2017 | Systolic blood pressure | NA | NA | 317754 | European | Males and Females | NA | https://gwas.mrcieu.ac.uk/datasets/ukb-a-360/ |
| ukb-a-359 | 2017 | Diastolic blood pressure | NA | NA | 317756 | European | Males and Females | NA | https://gwas.mrcieu.ac.uk/datasets/ukb-a-359/ |
| ieu-b-39 | 2018 | Diastolic blood pressure | NA | NA | 757601 | European | Males and Females | 30224653 | https://gwas.mrcieu.ac.uk/datasets/ieu-b-39/ |
| ebi-a-GCST005186 | 2012 | Fasting blood glucose | NA | NA | 58074 | European | NA | 22581228 | https://gwas.mrcieu.ac.uk/datasets/ebi-a-GCST005186/ |
| ebi-a-GCST000568 | 2010 | Fasting blood glucose | NA | NA | 46186 | European | NA | 20081858 | https://gwas.mrcieu.ac.uk/datasets/ebi-a-GCST000568/ |
| ieu-b-103 | 2010 | HbA1C | NA | NA | 46368 | European | Males and Females | 20858683 | https://gwas.mrcieu.ac.uk/datasets/ieu-b-103/ |
| ieu-b-4842 | 2022 | HbA1C | NA | NA | 45734 | European | Males and Females | NA | https://gwas.mrcieu.ac.uk/datasets/ieu-b-4842/ |
| ieu-b-4849 | 2022 | Triglycerides | NA | NA | 30515 | European | Males and Females | NA | https://gwas.mrcieu.ac.uk/datasets/ieu-b-4849/ |
| ieu-b-111 | 2020 | Triglycerides | NA | NA | 441016 | European | Males and Females | 32203549 | https://gwas.mrcieu.ac.uk/datasets/ieu-b-111/ |
| ebi-a-GCST005068 | 2017 | LDL cholesterol | NA | NA | 9961 | European | NA | 28887542 | https://gwas.mrcieu.ac.uk/datasets/ebi-a-GCST005068/ |
| ebi-a-GCST002222 | 2013 | LDL cholesterol | NA | NA | 94595 | European | NA | 24097068 | https://gwas.mrcieu.ac.uk/datasets/ebi-a-GCST002222/ |
| met-d-HDL_C | 2020 | HDL cholesterol | NA | NA | 115078 | European | Males and Females | NA | https://gwas.mrcieu.ac.uk/datasets/met-d-HDL_C/ |
| ieu-b-109 | 2020 | HDL cholesterol | NA | NA | 403943 | European | Males and Females | 32203549 | https://gwas.mrcieu.ac.uk/datasets/ieu-b-109/ |
