# Supplementary material for: Relationship of vegetarianism with body weight loss and ASCVD
Source: Front Nutr. 2024 Aug 27;11:1419743. doi: 10.3389/fnut.2024.1419743 (PMC11389726; doi:10.3389/fnut.2024.1419743)
Supplement: Supplementary file 1 [file Data_Sheet_1.zip › Table S5.DOCX]

| **id.exposure** | **id.outcome** | **outcome** | **exposure** | **egger_intercept** | **se** | **pval** |
| --- | --- | --- | --- | --- | --- | --- |
| ukb-b-1996 | ebi-a-GCST002783 | Body mass index \|\| id:ebi-a-GCST002783 | Salad / raw vegetable intake \|\| id:ukb-b-1996 | -0.002759455 | 0.010580165 | 0.801742349 |
| ukb-b-1996 | ieu-a-92 | Obesity class 3 \|\| id:ieu-a-92 | Salad / raw vegetable intake \|\| id:ukb-b-1996 | -0.018042947 | 0.069990351 | 0.800318239 |
| ukb-b-1996 | finn-b-I9_CHD | Major coronary heart disease event \|\| id:finn-b-I9_CHD | Salad / raw vegetable intake \|\| id:ukb-b-1996 | 0.03162341 | 0.018410672 | 0.104016782 |
| ukb-b-1996 | finn-b-I9_MI | Myocardial infarction \|\| id:finn-b-I9_MI | Salad / raw vegetable intake \|\| id:ukb-b-1996 | 0.014925641 | 0.024464565 | 0.549869925 |
| ukb-b-1996 | ebi-a-GCST005843 | Ischemic stroke \|\| id:ebi-a-GCST005843 | Salad / raw vegetable intake \|\| id:ukb-b-1996 | 0.003355805 | 0.017421162 | 0.849834012 |
| ukb-b-1996 | finn-b-I9_HYPTENSESS_EXNONE | "Hypertension, essential" (no controls excluded) \|\| id:finn-b-I9_HYPTENSESS_EXNONE | Salad / raw vegetable intake \|\| id:ukb-b-1996 | -0.004665084 | 0.013416303 | 0.732320378 |
| ukb-b-1996 | ieu-a-26 | Type 2 diabetes \|\| id:ieu-a-26 | Salad / raw vegetable intake \|\| id:ukb-b-1996 | 0.027573456 | 0.041322371 | 0.523372603 |
| ukb-b-1996 | ukb-b-17462 | Diagnoses - secondary ICD10: E78.5 Hyperlipidaemia, unspecified \|\| id:ukb-b-17462 | Salad / raw vegetable intake \|\| id:ukb-b-1996 | -0.0001023 | 0.000373376 | 0.787832496 |
| ukb-b-1996 | ukb-a-360 | Systolic blood pressure automated reading \|\| id:ukb-a-360 | Salad / raw vegetable intake \|\| id:ukb-b-1996 | 0.00016465 | 0.006664155 | 0.980614513 |
| ukb-b-1996 | ukb-a-359 | Diastolic blood pressure automated reading \|\| id:ukb-a-359 | Salad / raw vegetable intake \|\| id:ukb-b-1996 | -0.001976474 | 0.00851227 | 0.819527633 |
| ukb-b-1996 | ieu-b-4849 | Triglycerides \|\| id:ieu-b-4849 | Salad / raw vegetable intake \|\| id:ukb-b-1996 | 0.006097658 | 0.023150208 | 0.798903019 |
| ukb-b-1996 | met-d-HDL_C | HDL cholesterol \|\| id:met-d-HDL_C | Salad / raw vegetable intake \|\| id:ukb-b-1996 | -0.004102964 | 0.006279388 | 0.52223745 |
| ukb-b-1996 | ebi-a-GCST005068 | LDL cholesterol \|\| id:ebi-a-GCST005068 | Salad / raw vegetable intake \|\| id:ukb-b-1996 | -0.059984994 | 0.029705287 | 0.078138858 |
| ukb-b-1996 | ebi-a-GCST005186 | Fasting blood glucose \|\| id:ebi-a-GCST005186 | Salad / raw vegetable intake \|\| id:ukb-b-1996 | 0.006191277 | 0.006802889 | 0.37817497 |
| ukb-b-1996 | ieu-b-103 | HbA1C \|\| id:ieu-b-103 | Salad / raw vegetable intake \|\| id:ukb-b-1996 | 0.013036966 | 0.00808389 | 0.141266209 |
