# Supplementary material for: Relationship of vegetarianism with body weight loss and ASCVD
Source: Front Nutr. 2024 Aug 27;11:1419743. doi: 10.3389/fnut.2024.1419743 (PMC11389726; doi:10.3389/fnut.2024.1419743)
Supplement: Supplementary file 1 [file Data_Sheet_1.zip › Table S6.DOCX]

| **id.exposure** | **id.outcome** | **outcome** | **exposure** | **method** | **Q** | **Q_df** | **Q_pval** |
| --- | --- | --- | --- | --- | --- | --- | --- |
| ukb-b-1996 | ebi-a-GCST002783 | Body mass index \|\| id:ebi-a-GCST002783 | Salad / raw vegetable intake \|\| id:ukb-b-1996 | Inverse variance weighted | 11.33710161 | 8 | 0.183320249 |
| ukb-b-1996 | ieu-a-92 | Obesity class 3 \|\| id:ieu-a-92 | Salad / raw vegetable intake \|\| id:ukb-b-1996 | Inverse variance weighted | 17.33446908 | 15 | 0.299266125 |
| ukb-b-1996 | finn-b-I9_CHD | Major coronary heart disease event \|\| id:finn-b-I9_CHD | Salad / raw vegetable intake \|\| id:ukb-b-1996 | Inverse variance weighted | 19.59286361 | 18 | 0.356201082 |
| ukb-b-1996 | finn-b-I9_MI | Myocardial infarction \|\| id:finn-b-I9_MI | Salad / raw vegetable intake \|\| id:ukb-b-1996 | Inverse variance weighted | 21.06416852 | 18 | 0.276188041 |
| ukb-b-1996 | ebi-a-GCST005843 | Ischemic stroke \|\| id:ebi-a-GCST005843 | Salad / raw vegetable intake \|\| id:ukb-b-1996 | Inverse variance weighted | 25.10916166 | 16 | 0.067924014 |
| ukb-b-1996 | finn-b-I9_HYPTENSESS_EXNONE | "Hypertension, essential" (no controls excluded) \|\| id:finn-b-I9_HYPTENSESS_EXNONE | Salad / raw vegetable intake \|\| id:ukb-b-1996 | Inverse variance weighted | 17.14497959 | 18 | 0.513159303 |
| ukb-b-1996 | ieu-a-26 | Type 2 diabetes \|\| id:ieu-a-26 | Salad / raw vegetable intake \|\| id:ukb-b-1996 | Inverse variance weighted | 8.559016217 | 9 | 0.478934199 |
| ukb-b-1996 | ukb-b-17462 | Diagnoses - secondary ICD10: E78.5 Hyperlipidaemia, unspecified \|\| id:ukb-b-17462 | Salad / raw vegetable intake \|\| id:ukb-b-1996 | Inverse variance weighted | 22.34744145 | 16 | 0.132323226 |
| ukb-b-1996 | ukb-a-360 | Systolic blood pressure automated reading \|\| id:ukb-a-360 | Salad / raw vegetable intake \|\| id:ukb-b-1996 | Inverse variance weighted | 55.77806336 | 16 | 2.65E-06 |
| ukb-b-1996 | ukb-a-359 | Diastolic blood pressure automated reading \|\| id:ukb-a-359 | Salad / raw vegetable intake \|\| id:ukb-b-1996 | Inverse variance weighted | 91.23410507 | 16 | 1.48E-12 |
| ukb-b-1996 | ieu-b-4849 | Triglycerides \|\| id:ieu-b-4849 | Salad / raw vegetable intake \|\| id:ukb-b-1996 | Inverse variance weighted | 19.04461303 | 9 | 0.024816221 |
| ukb-b-1996 | met-d-HDL_C | HDL cholesterol \|\| id:met-d-HDL_C | Salad / raw vegetable intake \|\| id:ukb-b-1996 | Inverse variance weighted | 26.94960895 | 18 | 0.079945496 |
| ukb-b-1996 | ebi-a-GCST005068 | LDL cholesterol \|\| id:ebi-a-GCST005068 | Salad / raw vegetable intake \|\| id:ukb-b-1996 | Inverse variance weighted | 10.43017131 | 9 | 0.316795125 |
| ukb-b-1996 | ebi-a-GCST005186 | Fasting blood glucose \|\| id:ebi-a-GCST005186 | Salad / raw vegetable intake \|\| id:ukb-b-1996 | Inverse variance weighted | 21.23749956 | 15 | 0.129411216 |
| ukb-b-1996 | ieu-b-103 | HbA1C \|\| id:ieu-b-103 | Salad / raw vegetable intake \|\| id:ukb-b-1996 | Inverse variance weighted | 11.92976458 | 10 | 0.289784609 |
