# Supplementary figures and images for: Relationship of vegetarianism with body weight loss and ASCVD
Source: Front Nutr. 2024 Aug 27;11:1419743. doi: 10.3389/fnut.2024.1419743 (PMC11389726; doi:10.3389/fnut.2024.1419743)

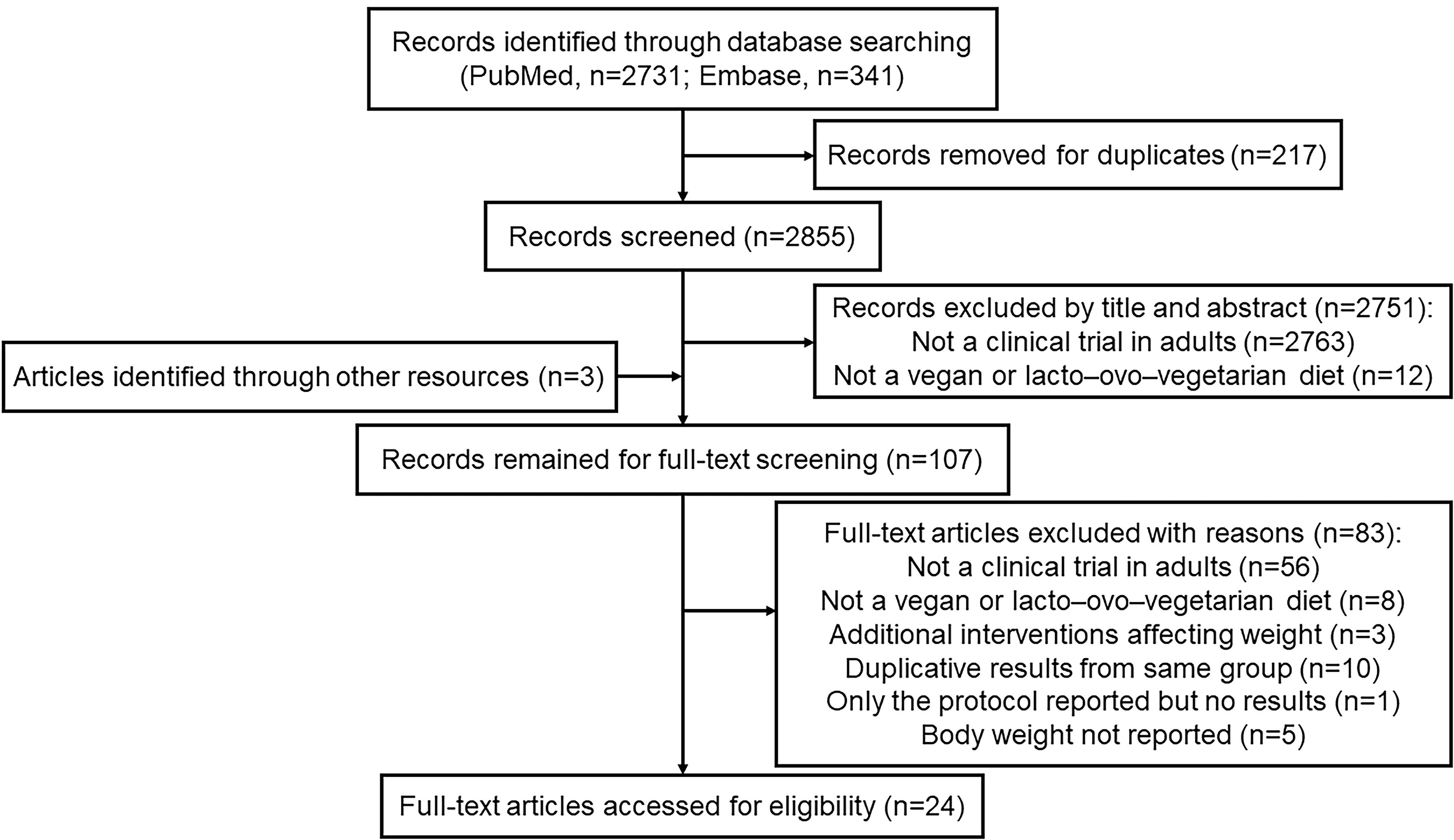

Supplement: Supplementary file 1 [file Data_Sheet_1.zip › Fig. S1.TIF]

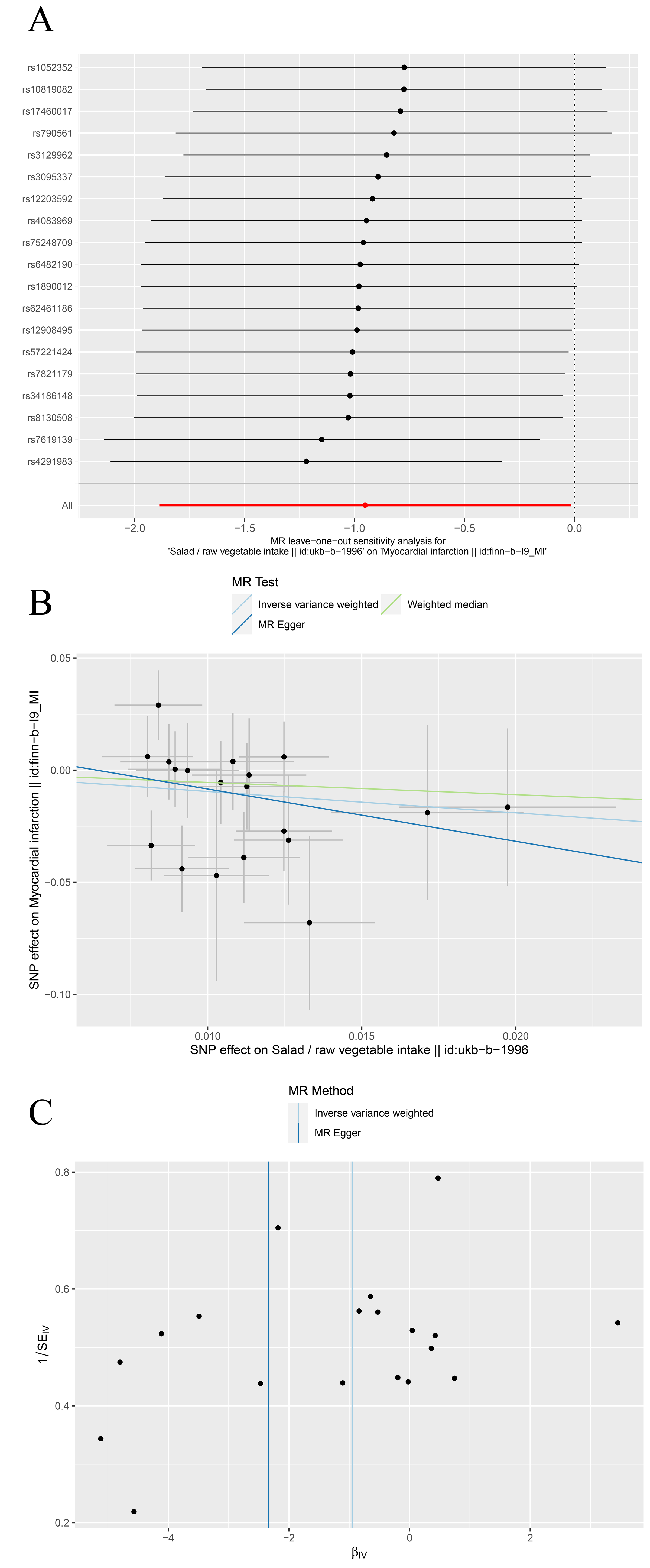

Supplement: Supplementary file 1 [file Data_Sheet_1.zip › Fig. S10.TIF]

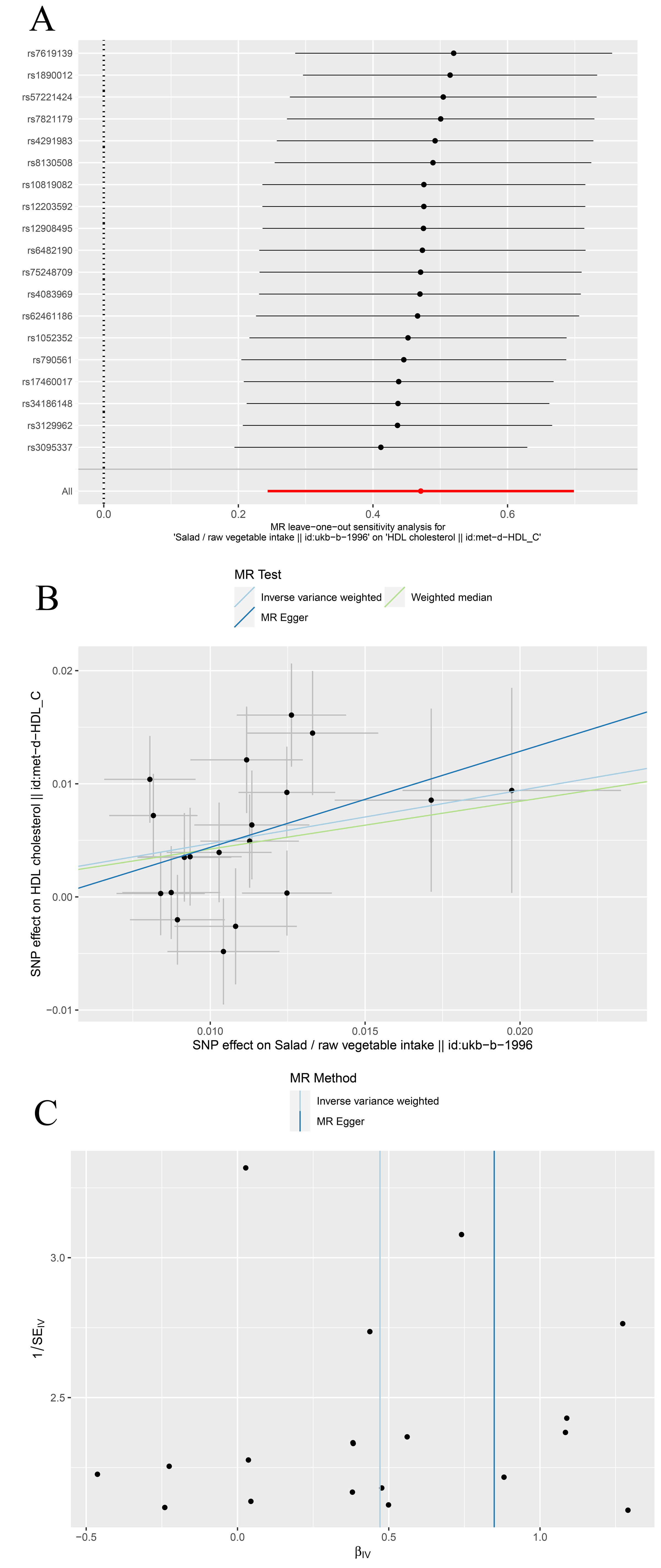

Supplement: Supplementary file 1 [file Data_Sheet_1.zip › Fig. S11.TIF]

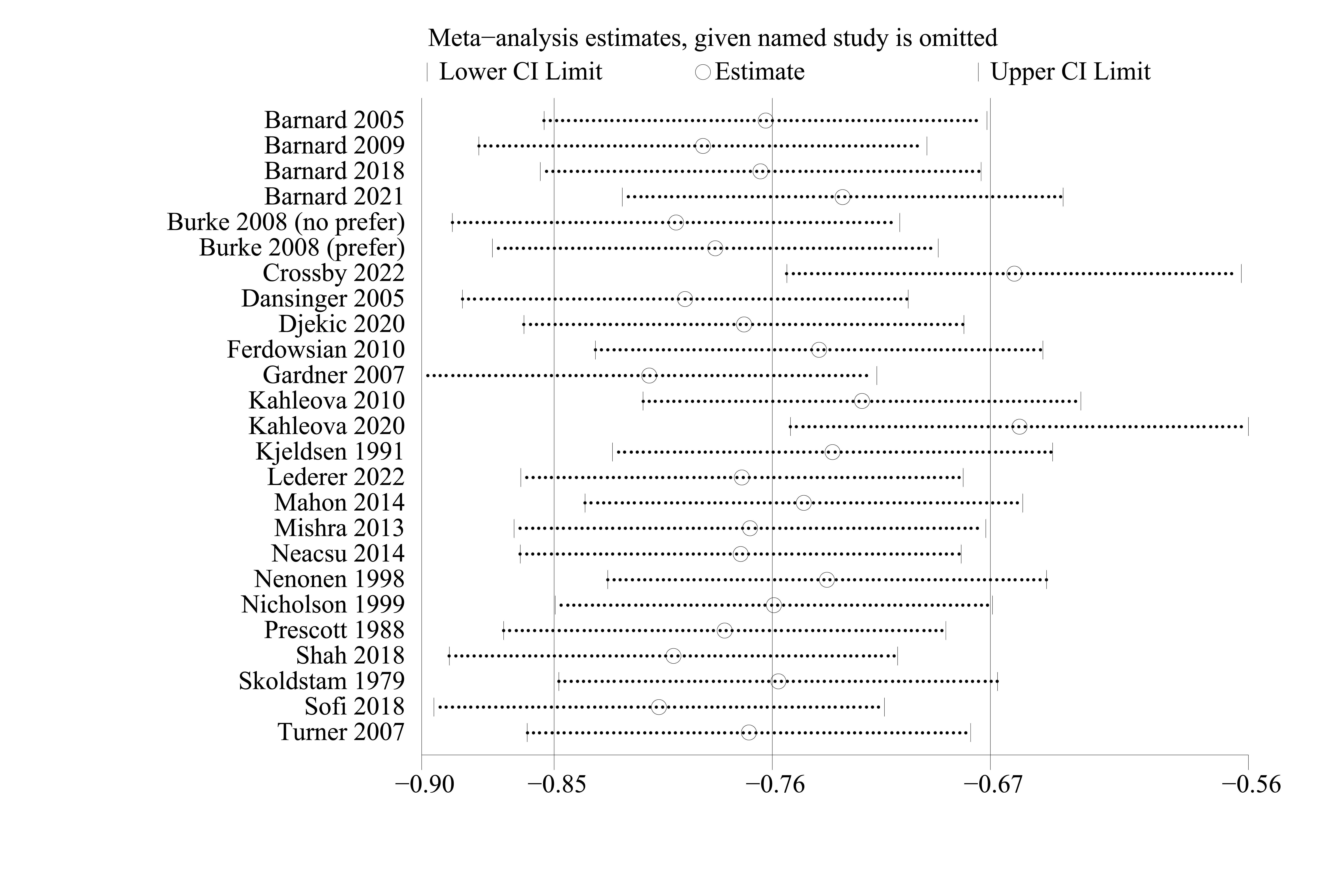

Supplement: Supplementary file 1 [file Data_Sheet_1.zip › Fig. S2.TIF]

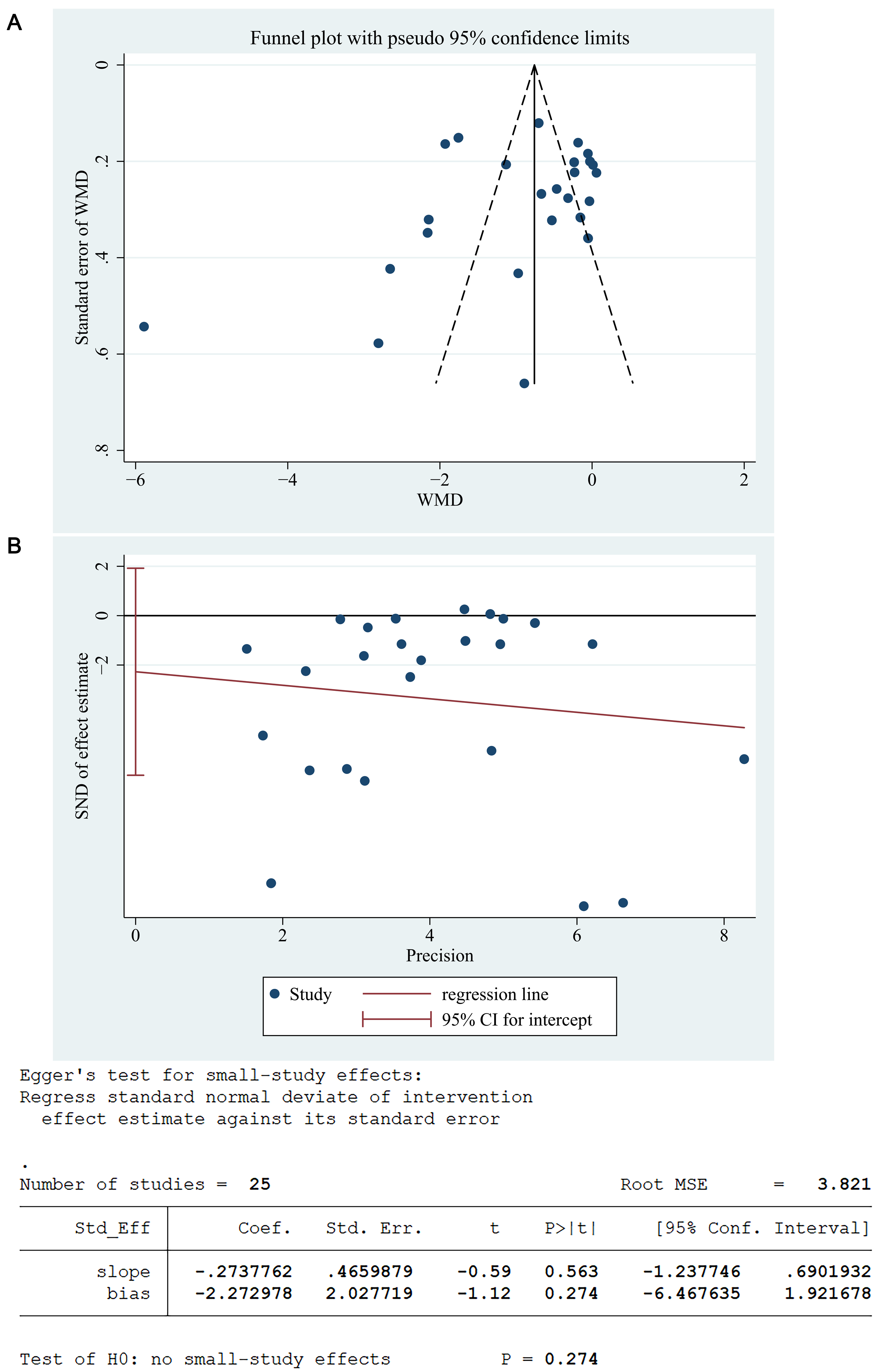

Supplement: Supplementary file 1 [file Data_Sheet_1.zip › Fig. S3.TIF]

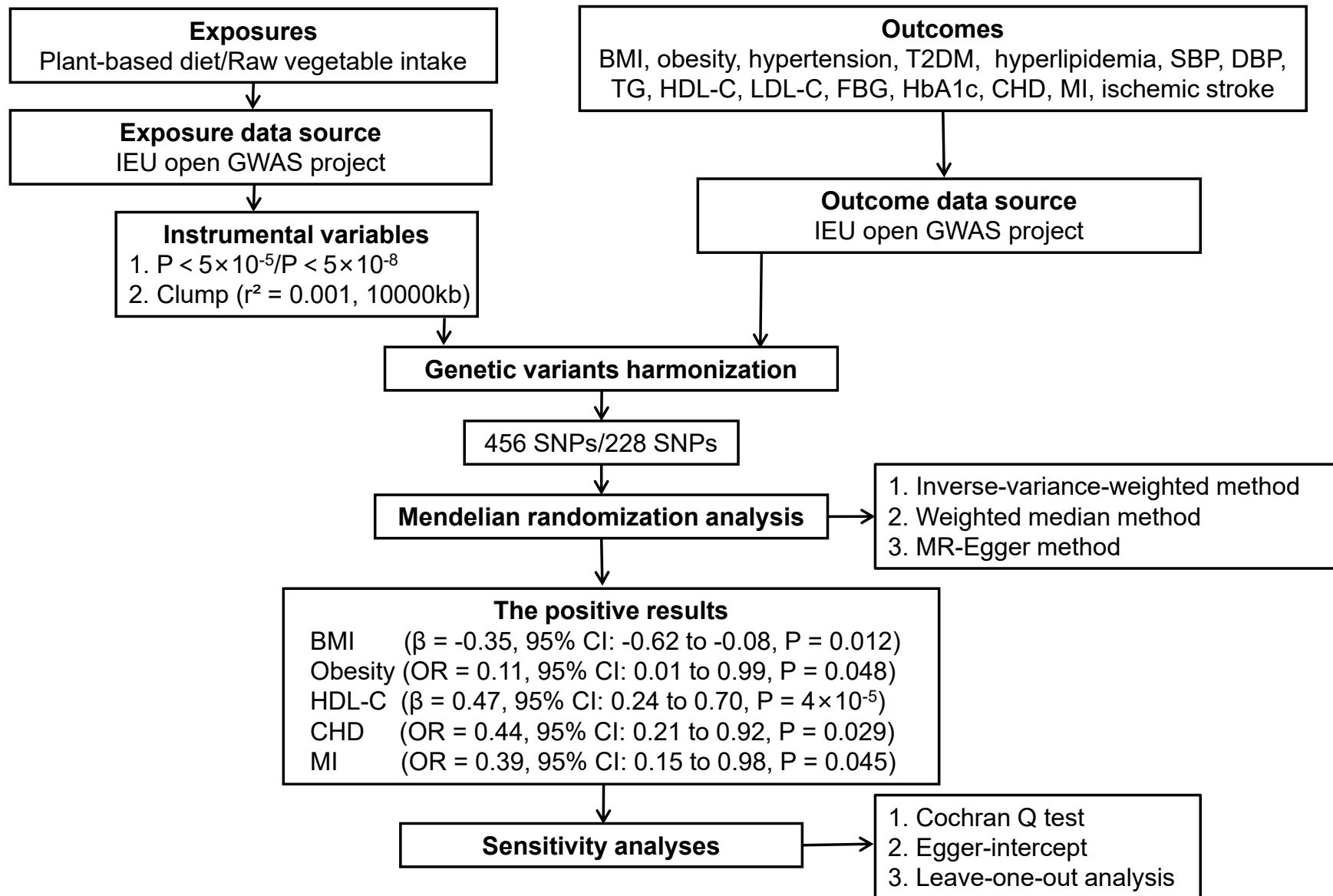

Supplement: Supplementary file 1 [file Data_Sheet_1.zip › Fig. S4.PDF]

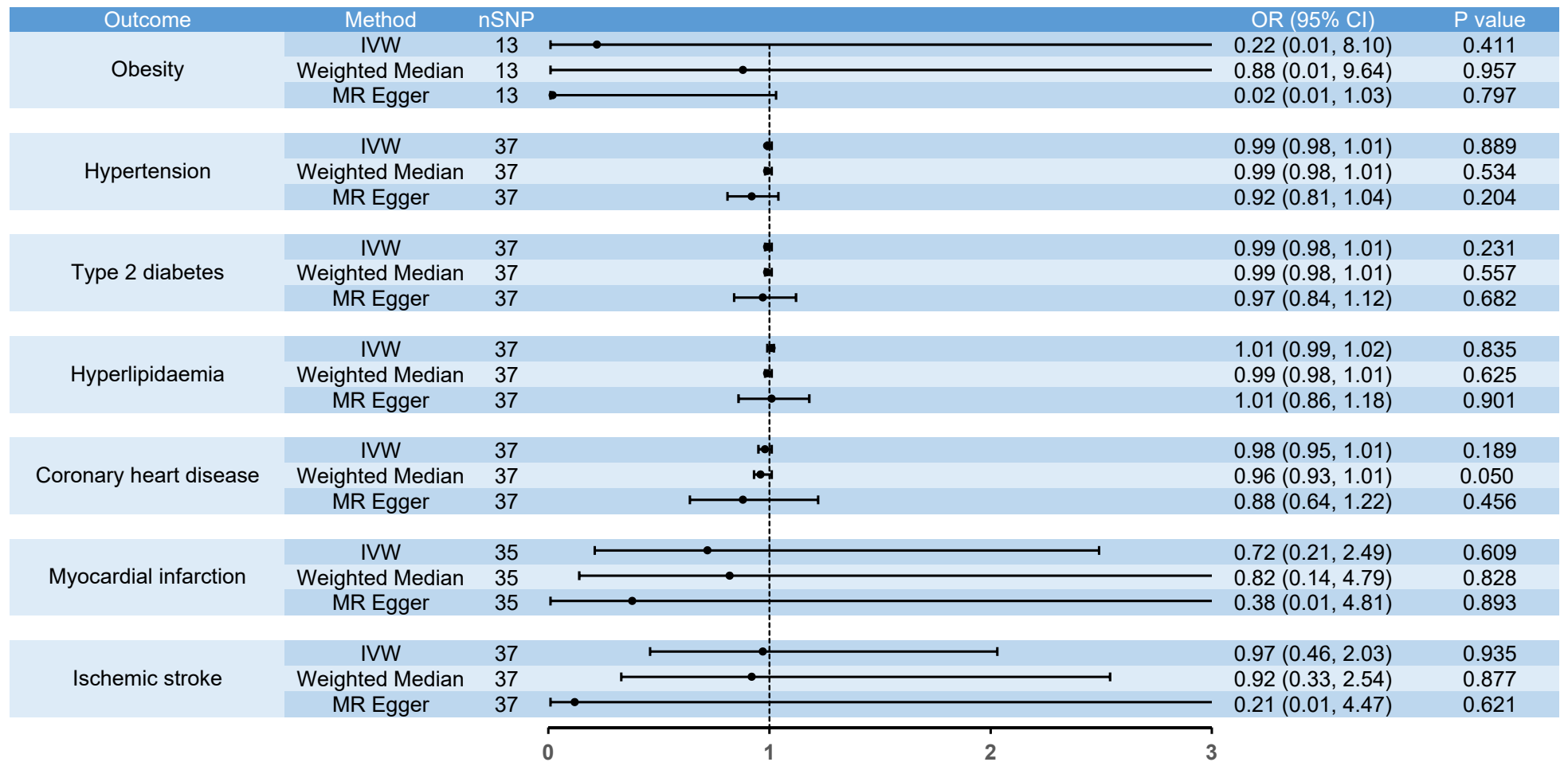

Supplement: Supplementary file 1 [file Data_Sheet_1.zip › Fig. S5.PDF]

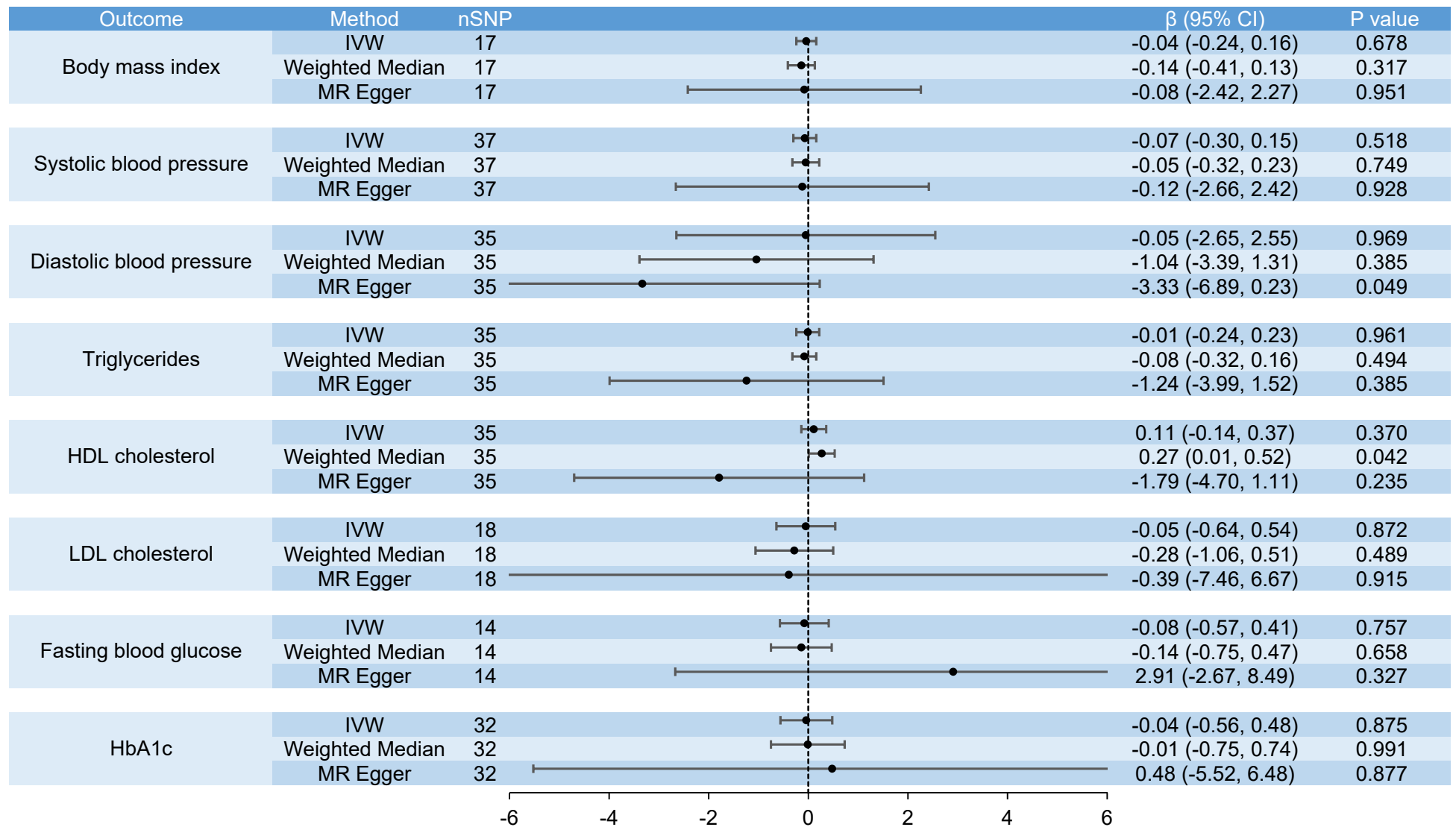

Supplement: Supplementary file 1 [file Data_Sheet_1.zip › Fig. S6.PDF]

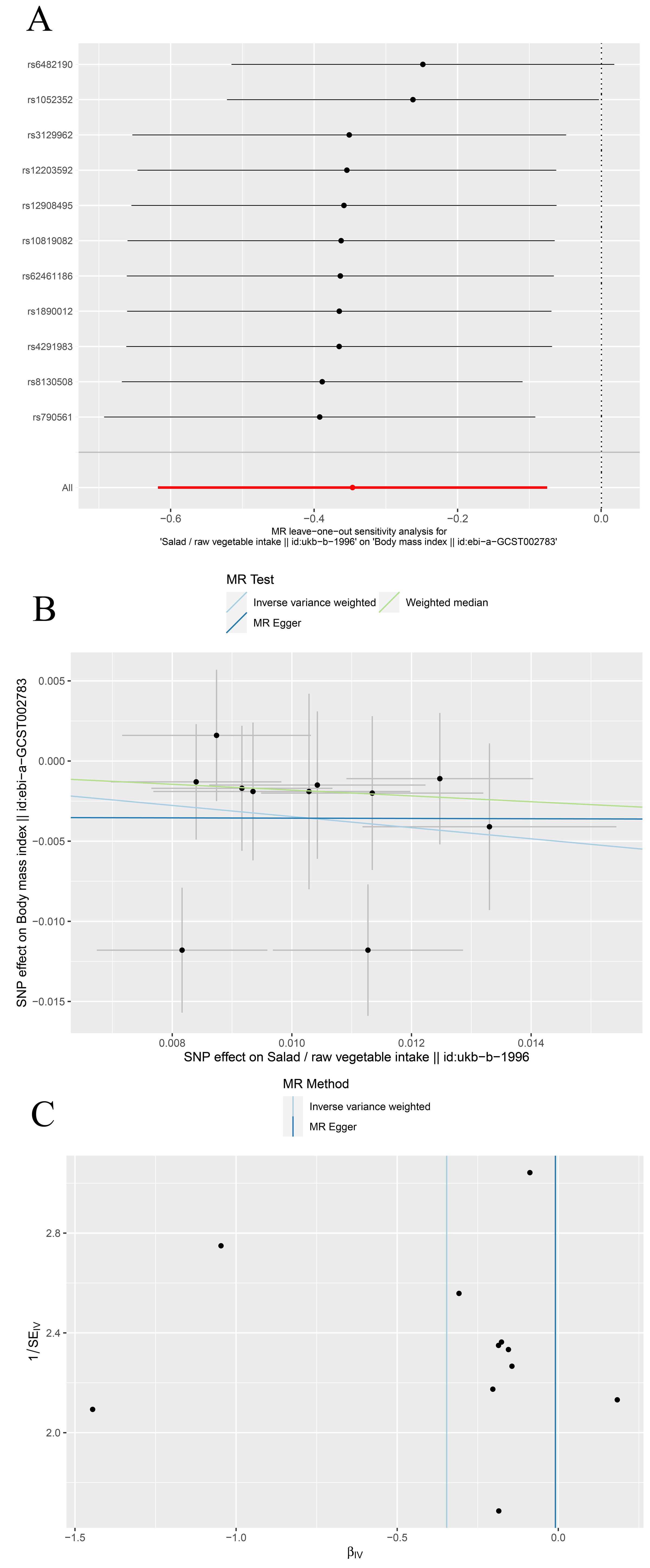

Supplement: Supplementary file 1 [file Data_Sheet_1.zip › Fig. S7.TIF]

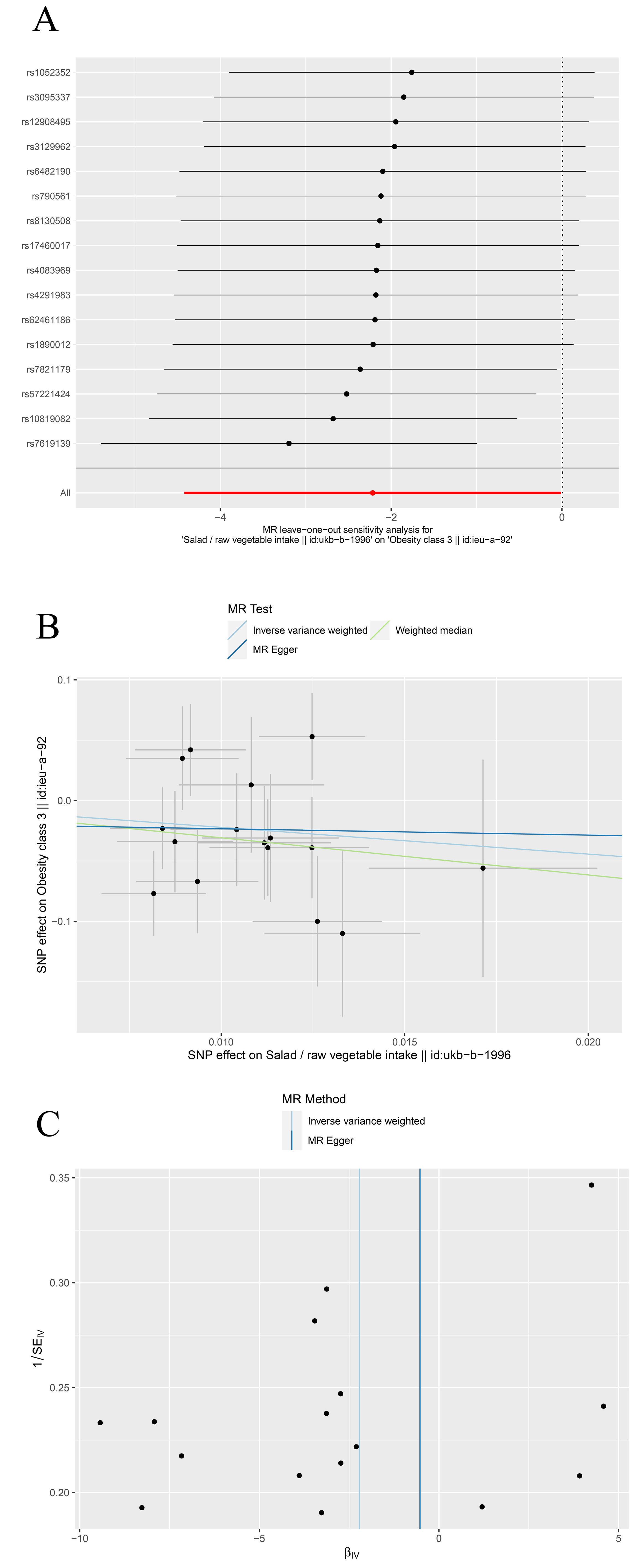

Supplement: Supplementary file 1 [file Data_Sheet_1.zip › Fig. S8.TIF]

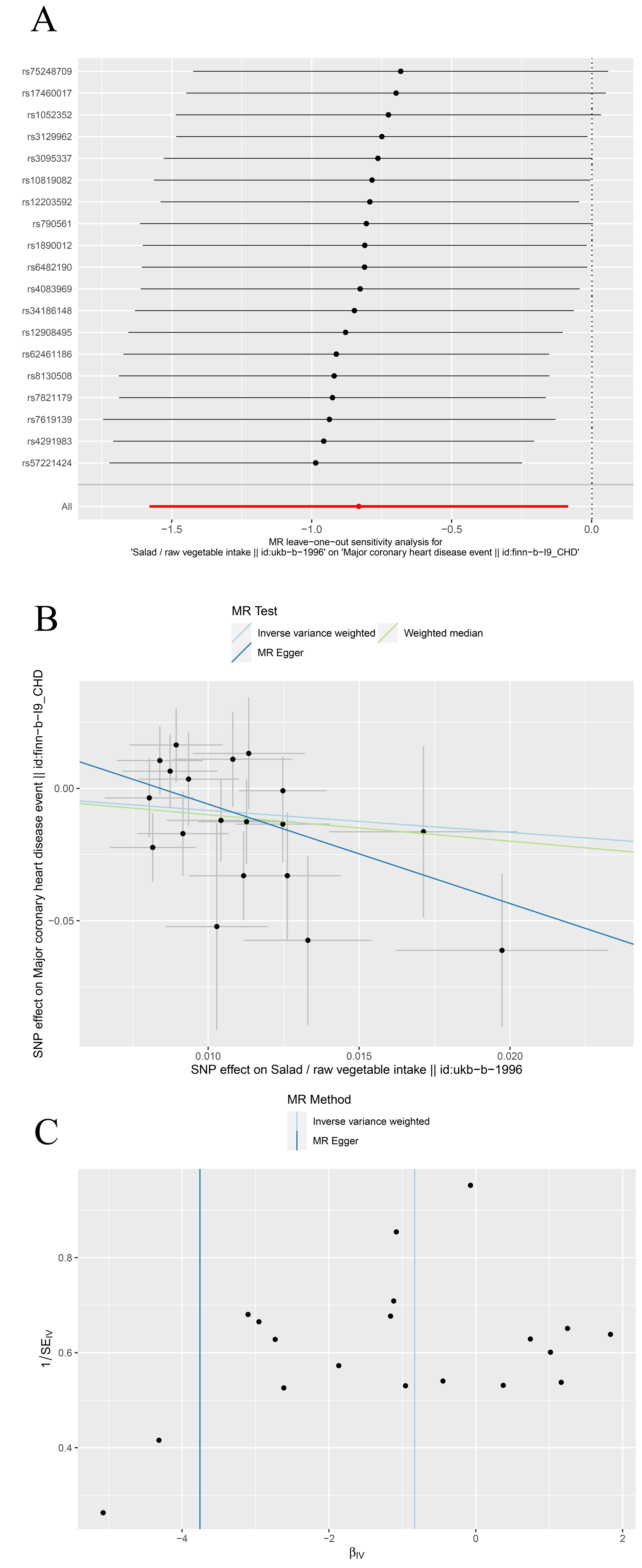

Supplement: Supplementary file 1 [file Data_Sheet_1.zip › Fig. S9.TIF]
